# Genome-wide association study of restless legs syndrome in end-stage renal disease

**DOI:** 10.1093/sleepadvances/zpag095

**Published:** 2026-08-29

**Authors:** Noah Risse, Philip Harrer, Giorgos K Sakkas, Ioannis Stefanidis, Nathalie Schandra, Ambra Stefani, Magdalena Wildt, Yves Dauvilliers, Georgios M Hadjigeorgiou, Chen Zhao, Juliane Winkelmann, Konrad Oexle, Barbara Schormair

**Affiliations:** Helmholtz Zentrum München, German Research Center for Environmental Health, Institute of Neurogenomics, Neuherberg, Germany; Institute of Human Genetics, TUM School of Medicine and Health, Technical University of Munich, Munich, Germany; Helmholtz Zentrum München, German Research Center for Environmental Health, Institute of Neurogenomics, Neuherberg, Germany; Institute of Human Genetics, TUM School of Medicine and Health, Technical University of Munich, Munich, Germany; Department of Physical Education and Sport Science, University of Thessaly, Trikala, Thessaly 42100, Greece; Department of Life Sciences, School of Life and Health Sciences, University of Nicosia, Nicosia, 2417, Cyprus; Department of Nephrology, School of Medicine, University of Thessaly, Larisa, Greece; Helmholtz Zentrum München, German Research Center for Environmental Health, Institute of Neurogenomics, Neuherberg, Germany; Institute of Human Genetics, TUM School of Medicine and Health, Technical University of Munich, Munich, Germany; Department of Neurology, Medical University of Innsbruck, Innsbruck, Austria; Department of Neurology, Medical University of Innsbruck, Innsbruck, Austria; Sleep–Wake Disorders Centre, Department of Neurology, Gui de Chauliac Hospital, Montpellier, France; Department of Neurology, Nicosia General Hospital Medical School, University of Cyprus, Nicosia, Cyprus; Helmholtz Zentrum München, German Research Center for Environmental Health, Institute of Neurogenomics, Neuherberg, Germany; Institute of Human Genetics, TUM School of Medicine and Health, Technical University of Munich, Munich, Germany; Helmholtz Zentrum München, German Research Center for Environmental Health, Institute of Neurogenomics, Neuherberg, Germany; Institute of Human Genetics, TUM School of Medicine and Health, Technical University of Munich, Munich, Germany; Munich Cluster for Systems Neurology (SyNergy), Munich, Germany; German Center for Mental Health (DZPG), partner site Munich–Augsburg, Munich–Augsburg, Germany; Helmholtz Zentrum München, German Research Center for Environmental Health, Institute of Neurogenomics, Neuherberg, Germany; Institute of Human Genetics, TUM School of Medicine and Health, Technical University of Munich, Munich, Germany; Neurogenetic Systems Analysis Group, Institute of Neurogenomics, Helmholtz Zentrum München, German Research Center for Environmental Health, Neuherberg, Germany; Helmholtz Zentrum München, German Research Center for Environmental Health, Institute of Neurogenomics, Neuherberg, Germany; Institute of Human Genetics, TUM School of Medicine and Health, Technical University of Munich, Munich, Germany

**Keywords:** restless legs syndrome, RLS, genome-wide association study, GWAS, genetic risk score, PRS, chronic renal diseases, ESRD

## Abstract

**Study Objectives:**

Restless legs syndrome (RLS) is predominantly idiopathic (iRLS), with a prevalence of 2%–10% in Europe. In end-stage renal disease patients (ESRD), uremic RLS (uRLS) is substantially more prevalent, with estimates of 24%–28%. Its pathophysiology has not been fully uncovered. Therefore, we investigated potential genetic and phenotypic risk factors for uRLS.

**Materials and Methods:**

We performed case–control genome-wide association studies (GWAS) on ESRD patients with and without RLS from two German and one Greek ESRD study populations (cases/controls: 186/356, 80/176, 134/357). We applied a polygenic risk score (PRS) for iRLS to four groups: uRLS cases, uremic controls, a group of iRLS cases and European 1000G individuals. Additionally, we investigated RLS associations with phenotype data available in one German dataset.

**Results:**

No SNP reached genome-wide significance in any single dataset or meta-analyses. The mean iRLS PRS was significantly higher in uRLS cases compared to uremic controls (*p* = 9.47e-10) and European 1000G individuals (*p* = 9.93e-6) and significantly smaller compared to iRLS cases (*p* = 4.84e-5). Among 164 available lead SNPs at known RLS risk loci, rs113851554 at the MEIS1 locus, the strongest known iRLS-associated variant, was significant after Bonferroni correction (0.05/164 = 3.05e-4) in the German uremic meta-analysis (*p* = 5.73e-5). Two loci rs36127502 and rs5769388, previously not associated with RLS, show suggestive significance (*p* = 1.37e-7, *p* = 1.92e-7) in a meta-analysis combining the three GWAS datasets. Regular smoking and parathormone levels were nominally associated with RLS in ESRD.

**Conclusions:**

Our findings indicate that uRLS and iRLS share a substantial part of their genetic architecture, while also pointing to candidate signals specific to uRLS.

## Graphical Abstract

**Graphical Abstract:**
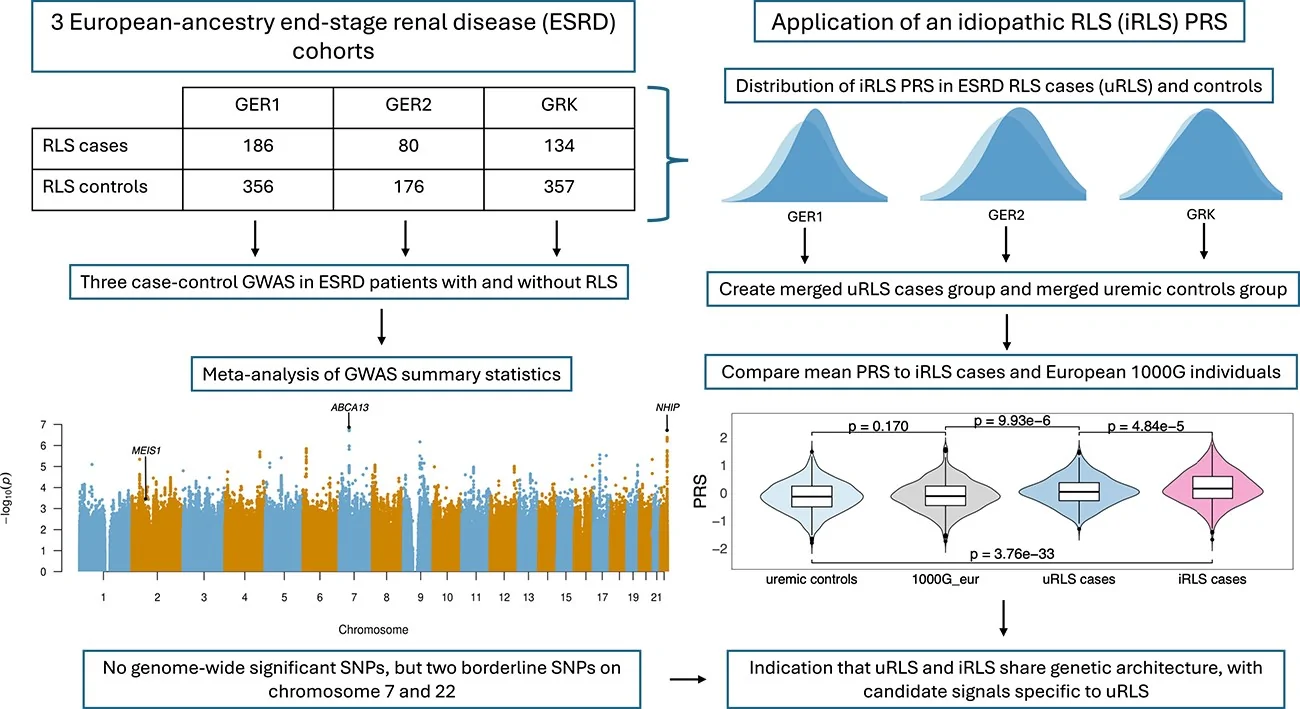

**Statement of Significance**

Restless legs syndrome (RLS) in end-stage renal disease (ESRD), termed uremic RLS, places a severe additional burden on the affected patients. Although substantially more prevalent than idiopathic RLS in the general population, its molecular etiology is not fully uncovered. In this exploratory study, we investigated candidate genetic and phenotypic risk factors to advance understanding of uremic RLS pathophysiology and conducted the first genome-wide association study on RLS in ESRD patients. The application of a polygenic risk score for idiopathic RLS to ESRD patients with and without RLS and idiopathic RLS cases indicated a shared genetic component of RLS in ESRD and non-ESRD RLS patients. We found two loci with suggestive associations that have not been associated with RLS previously.

## Introduction

Individuals diagnosed with RLS suffer with varying severity from a recurring urge to move their extremities due to unpleasant sensations that occur during periods of rest [1]. Sleep and quality of life of affected individuals are impaired. However, this sensorimotor disorder goes frequently undiagnosed. The pathophysiology has not been fully elucidated, but converging evidence from neuroimaging, genetic, and pharmacological studies implicates brain iron deficiency, dopaminergic dysfunction, as well as adenosinergic and glutamatergic disturbance [1]. RLS has a substantial heritable component with heritability estimates of 54.0 and 69.4% [2]. The prevalence of RLS varies across populations worldwide, being low in Asia, for instance, but as high as 5.9% in Europe [3]. Among European dialysis patients, the prevalence is substantially increased with estimates around 24% [4]. Furthermore, among patients with chronic kidney disease, the prevalence of RLS is substantially higher in patients with ESRD undergoing dialysis (28%) than in earlier stages of kidney disease (10%) [5]. Even though ESRD and its associated metabolic changes can be considered strong drivers of the risk to develop uremic RLS, this does not preclude a contribution from genetic susceptibility. The determinants of why only a subset of dialysis patients suffers from RLS remain poorly understood and genetic susceptibility could play a role. However, genetic studies of uRLS are scarce and have been limited to selected variants previously associated with idiopathic RLS, some of which also showed significant associations with uRLS [6, 7]. By focusing only on a subset of variants, these studies did not fully capture the polygenic architecture of the disease. Therefore, we decided to investigate the genetic basis of uRLS on a broader scale. We conducted, to our knowledge, the first GWAS of RLS in European-ancestry ESRD patients. Hereby, we investigated if there are variants that may exert effects specifically in the context of renal disease and whether genetic variants commonly associated with iRLS also show associations with uRLS in the available data. Furthermore, we applied a iRLS derived PRS [8] to uRLS cases, uremic patients without RLS, idiopathic RLS cases and the European 1000G individuals to investigate the degree to which the genetic liability for iRLS captured by the PRS is also present in uRLS. For a subgroup of the German patients, non-genetic data like BMI, diabetes status, and laboratory parameters were available and assessed for their role as potential risk factors of RLS.

## Materials and methods

### Data

#### Study populations

The first set of German dialysis patients (GER1) was recruited from 16 dialysis centers in Munich and the surrounding region between 2005 and 2008 and consists of 542 individuals. The Greek set of 491 dialysis patients (GRK) was recruited between 2007 and 2009 from seven dialysis centers in central and northern Greece. A third set (GER2) consists of 256 dialysis patients recruited in 2013 in seven dialysis centers located in Munich and the surrounding area. For all datasets, the final RLS diagnosis was established in face-to-face interviews based on the IRLSSG diagnostic criteria [6, 9]. For GER2, additional phenotypic data was available: sex, age, smoking status (regular vs non regular), alcohol consumption (regular vs non regular), BMI, diabetes, ferritin (ng/ml), transferrin saturation (%), hemoglobin (g/dL), hematocrit (%), cholesterol (mg/dl), calcium (mmol/l), phosphate (mg/dl), parathormone (pmol/l), C-reactive protein (CRP; mg/l), leucocytes (giga/l), HbA1c (%), thyroidstimulating hormone (TSH; mU/l), and dialysis parameters: dialysis dosage (hours per week), dialysis method (hemodialysis [HD], hemodiafiltration [HDF], or hemofiltration [HF]), dialysis vintage (total length of time a patient has been on dialysis, in months), urea reduction ratio (URR; quantifying dialysis treatment adequacy, has no unit) and Kt/V (quantifying dialysis treatment adequacy, has no unit). Additionally, a set of idiopathic RLS cases (iRLS cases) consisting of 949 patients recruited in France, Austria, and Germany was included for PRS comparison. RLS was diagnosed in face-to-face interviews based on the IRLSSG diagnostic criteria [9]. Information on age and sex distributions in the cohorts can be found in Table 1.

**Table 1:** Demographic and clinical information on data sets GER1, GER2, GRK, and iRLS cases

	GER1	GER2	GRK	iRLS cases
RLS (cases/controls)	186/356	80/176	134/357	949/0
Sex in RLS cases (m/f/u)	105/81/0	55/25/0	77/57/0	404/544/1
Sex in RLS controls (m/f/u)	228/128/0	125/51/0	216/141/0	-
Age (median, [IQR])	66 [58, 74]	67 [54, 77]	67 [57, 74]	-
Age in RLS cases (median, [IQR])	66 [56.25, 74]	66 [54, 76]	61.5 [53, 68]	60 [50.5, 72]^*^
Age in RLS controls (median, [IQR])	66 [58, 74]	67 [54, 77]	69 [60, 75]	-

The abbreviations m, f, u, and IQR stand for male, female, unknown, and inter quartile range, respectively.

^*^Information was available only for 319 of 949 individuals.

#### Genotyping data

Individuals of GER1 were genotyped on the Affymetrix Axiom array as part of a larger genotyping project [10] and a quality control (QC) on the genotype data was performed in that project. The present study included 542 of the 551 patients from the first German set [10]. The dialysis patients of GRK were genotyped on the Illumina GSA array together with individuals of GER2. Furthermore, the individuals of the dataset iRLS cases were also genotyped on the Illumina GSA array.

### Analysis of genetic data

#### Quality control and imputation

To the genetic data of the four data sets GER1, GER2, GRK, and iRLS cases a standard QC was applied using PLINK 1.9 [11, 12]: Variants were excluded if they had a call rate of less than 98%, a minor allele frequency smaller than 0.01 or if they significantly deviated from the Hardy–Weinberg-Equilibrium with a significance of less than *p* = 1e-10. To assess genetic ancestry, the individuals of each GWAS dataset were projected onto a multidimensional scaling (MDS) components plot using the 1000G data, which be found in Figure S3. Individuals were removed from the analysis if they failed PLINK’s sex check, had a call rate of less than 98%, deviated more than 3 SD from the heterozygosity mean of the samples of the respective data set, or deviated more than 3 SD from the mean of either of the first 5 MDS components within each data set. Additionally, in-data set relatedness was assessed using a PIHAT value of 0.2. For related pairs, the individual with the lower call rate was removed. For further analysis non-autosomal SNPs were removed. After QC the data sets were separately imputed with the HRC reference panel using the Helmholtz Munich Imputation Server (https://imputationserver.helmholtz-munich.dehttps://imputationserver.helmholtz-munich.de/) [13]. Before calculating associations between SNPs and RLS status, relatedness was assessed between GER1 and GER2, leading to the removal of 13 duplicates and one sibling from GER1. The remaining sample sizes in each data set after QC were 169 RLS cases and 332 uremic controls for GER1, 74 RLS cases, and 164 RLS controls for GER2, 118 RLS cases, and 319 uremic controls for GRK and 875 iRLS cases.

#### GWAS and meta-analyses

In order to use genotype probabilities instead of estimated hard call genotypes for SNP-RLS association the software SNPTEST (version 2.5.6) was used. Age, sex, and the first five principal components (PCs) based on the within data set similarity matrix for each uremic data set were included. Scree plots for each uremic data set (see Figure S4) showed an elbow after the first or second PC, respectively. We included five PCs as a conservative measure to control for potential residual population structure, in line with GWAS conventions [14]. Only SNPs with a Minimac info score (R2) larger than 0.3 and with a minor allele count of at least 20 in each dataset were assessed for SNP-RLS association. Fixed-effect meta-analyses of the summary statistics of all three dialysis cohorts (abbr: MetaALL) and of the two German dialysis cohorts only (abbr: MetaGER) were performed using METAL (release 2011-03-25) [15]. As in [6] we compared the results of the German study population to the Greek study population and then to the results of the combined study population. We decided to combine both German study populations into MetaGER, to increase power as the sample sizes are limited.

#### PRS selection

To further investigate the genetic make-up of uremic RLS and to compare it to idiopathic RLS a RLS PRS which is based on the largest meta-analysis of RLS GWAS to date [8] was applied to the cohorts of this study. Training of that RLS PRS did not include any individual from the four data sets GER1, GER2, GRK, or iRLS cases. As discussed in [8], RLS comorbid conditions include psychiatric disorders, cardiovascular disorders, hypertension and metabolic conditions. Representative PRS for each of these 4 conditions were selected from Polygenic Score Catalog (https://www.pgscatalog.orghttps://www.pgscatalog.org/). Candidate PRS had to be based on European discovery data and those with the largest discovery sets were chosen. If more than one trait-specific PRS were left after this selection, the one with the best performance metrics was chosen. Thus, PRS for the following traits were applied: depression (PGS003333), coronary artery disease (PGS004595), hypertension (PGS002765), type 2 diabetes (T2D; PGS000805), heart failure (PGS003969), ischemic stroke (PGS000911), abdominal aortic aneurism (PGS000753), low density lipoprotein cholesterol measurement (PGS003976) and anxiety (PGS004451).

#### PRS calculation

In order to see how each PRS performs in the average population compared to uremic RLS and the idiopathic RLS cohort, the PRS were also applied to the 503 European (CEU, FIN, GBR, IBS, TSI) 1000G samples (1000G eur) [16]. To deal with PRS SNP missingness in the five data sets the following strategy was applied: A SNP that is needed for the calculation of a PRS was considered missing either if it was not imputed in each and every data set or if the imputation score R^2^ was below 0.4 in any of the data sets, as proposed in [17]. This was done to ensure comparability between data sets. To maximize power for smaller sized PRS the following distinction was made: If the count of missing SNPs was less than 5000 and the percentage of missing SNPs was more than 5% , a proxy procedure was applied. For a missing SNP the procedure begins by calculating a list of SNPs that are in linkage disequilibrium with the SNP based on the 1000G samples of European descent. All SNPs that have a correlation coefficient of at least 0.6 with the missing SNP are considered. In the next step only those candidate SNPs are kept, that appear in all datasets with an imputation score R^2^ of at least 0.4. If there are still candidates left, the final proxy gets chosen from the candidates using the correlation coefficient from the first step: The candidate proxy with the largest correlation to the missing SNP is chosen as the final proxy. Thereafter, the weight for the PRS calculation incorporating the proxy SNP is calculated by multiplying the PRS weight of the missing SNP with its correlation coefficient with the chosen proxy in order to account for the degree of linkage disequilibrium. To account for possible ancestry bias in PRS, we calculated the first five principal components (PC) from the combined genotyping data of all 5 datasets restricted to HapMap SNPs, and regressed the raw PRS results onto the PCs. The residuals of this regression were then used as an ancestry adjusted PRS.

#### PRS analysis

We investigated mean differences of adjusted PRS between the groups 1000G eur, uremic controls (combining uremic controls from GER1, GER2, and GRK), uRLS cases (combining RLS cases from GER1, GER2, and GRK) and iRLS cases for each trait. Normality of adjusted PRS of each group was assessed using Shapiro–Wilk test and Q-Q plots. A one-way analysis of variance (ANOVA) without assuming equality of variances was applied. If a result was significant with respect to the significance level *α* = 0*.*05, we conducted post-hoc pairwise Welch’s *t*-tests. As data regarding sex and age was fully available only for the uremic data sets, we implemented an additional sensitivity analysis for the comparison of uremic controls versus uremic RLS cases. Hereby we fitted for each uremic data set a linear regression model with adjusted PRS as outcome and RLS status, age, and sex as covariates. The effect estimates for RLS status were combined across datasets using a random-effects meta-analysis with restricted maximum likelihood (REML) estimation. Detailed summary statistics of all tests and for every PRS can be found in Notes S1–S11. Furthermore, we compared PRS of uRLS cases with the subset of 306 iRLS cases for which the age was available after genotype quality control. Hereby we fitted a linear regression model with adjusted PRS as outcome and RLS type (uremic or idiopathic), age and sex as covariates to investigate whether sex and age differences between uRLS cases and iRLS cases could explain a difference between mean PRS in these two groups. Additionally, we checked whether the results of the pairwise comparisons of the RLS PRS could be driven by ESRD patients with positive family history of RLS. Specifically, 21 RLS cases with confirmed familial RLS in GER1, 8 RLS cases, and 3 controls with confirmed familial RLS in GER2, and 6 RLS cases with confirmed familial RLS in GRK were excluded for a robustness check. Detailed information on familial RLS in the uremic datasets can be found in Table S5. In the computational workflow of this study GNU Parallel was used for parallel execution of tasks [18].

### Analysis of phenotypic data of GER2

For each continuous variable of the available phenotypic data of GER2 the median and the inter quartile range (IQR) between the third and the first quartile was calculated for uremic controls, RLS cases and both groups combined. For dichotomous variables the total number of samples in RLS cases, controls and both groups combined are provided in Table 6. A first screening for association between RLS status and each single variable was done using univariate logistic regression models. In order to control for confounding, a combined logistic regression model was employed as well. The sample size of 80 RLS cases and 176 uremic controls limits the statistical power for a combined model when including 23 variables and over-fitting could become a problem. Therefore, a selection criterion was applied to choose those variables which showed a *p*-value *< .*2 in the univariate analysis. Using logistic regression models for the univariate tests instead of association tests like chi-squared tests or t-tests, ensures consistency in modeling when using the results as a criterion for variable inclusion into the combined model.

## Results

### GWAS and meta-analyses

**Figure 1:**
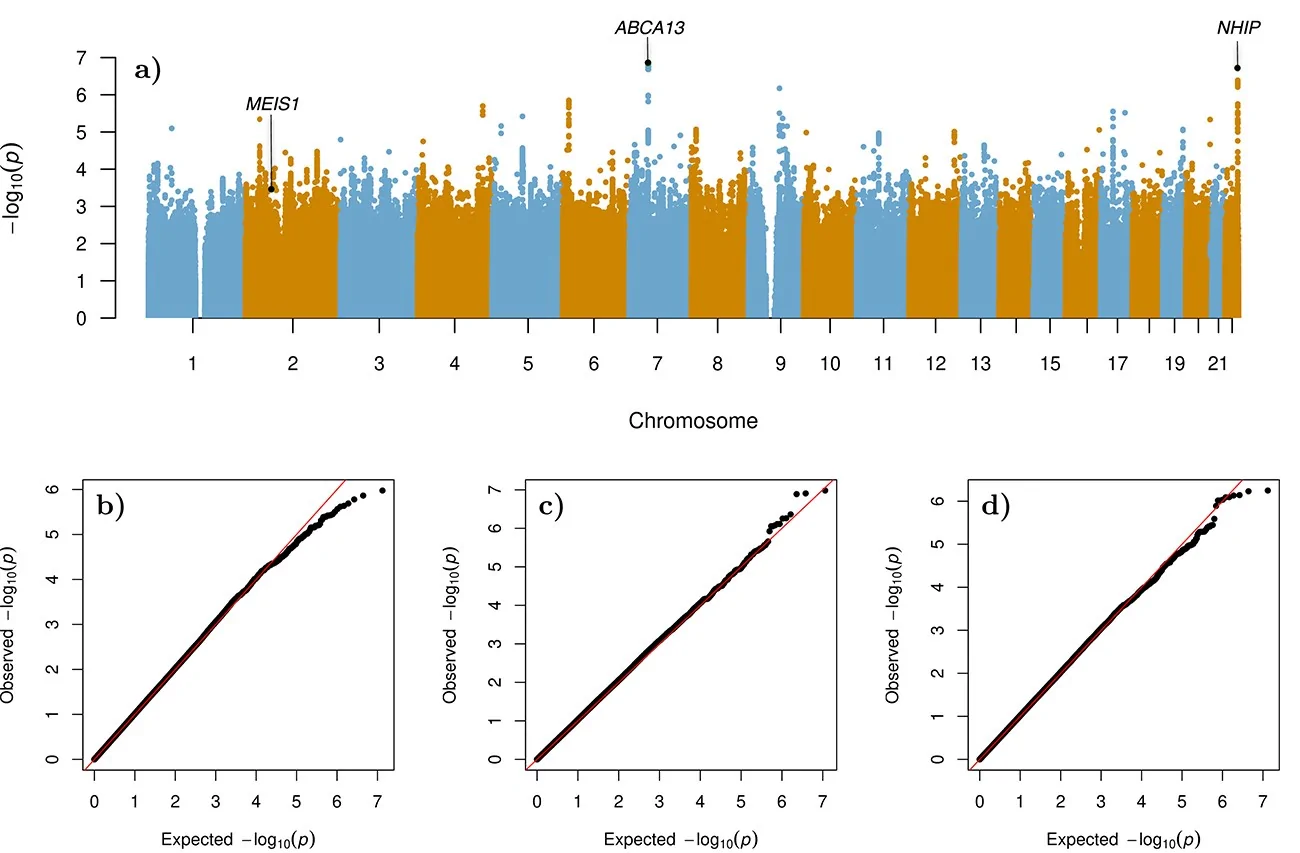
(a) Manhattan plot of MetaAll summary statistics. The three marked loci correspond to rs113851554, rs36127502, and rs5769388. Loci are labeled by the gene containing the variant or for rs5769388, by *NHIP*. (b–d) Q–Q plots for GER1, GER2, and GRK, respectively.

GWAS summary statistics did not show genome-wide significant associations with RLS status (*p <* 5e-8) in all three uremic datasets. The same was true for the meta-analyses MetaALL and MetaGER. Of all 196 independent RLS GWAS lead SNPs at 164 risk loci identified by Schormair et al. [8], summary statistics were derived for 165 SNPs in MetaALL, 164 SNPs in MetaGER, and 163 SNPs in GRK. The difference in variant availability can be attributed to the separate imputation of the datasets. For these available SNPs, we found nominal significance (*p* = .05) for 12 SNPs in MetaGER (Table 2), 8 SNPs in summary statistics of GRK (Table 3) and 14 SNPs in MetaALL (Table 4). Within the sets of nominal significant SNPs, GRK and MetaGER had no overlap, GRK and MetaALL an overlap of two SNPs, and MetaGER and MetaALL an overlap of eight SNPs. Effect directions were consistent with those reported in [8] for all nominally significant SNPs in both MetaGER and MetaALL, except for two SNPs—rs851663 and rs2748809—which showed discordant directions. Also, the SNPs rs2914339 and rs1326611, which show nominal significance in GRK show discordant directions. We applied Bonferroni correction based on the subset of the 196 lead SNPs identified in [8] that were available for testing in the two meta-analyses and the Greek GWAS, respectively. Hence, the significance thresholds amounted to 0.05/164 = 3.05e-4 for MetaGER, 0.05/165 = 3.03e-4 for MetaALL and 0.05/163 = 3.07e-4 for the Greek GWAS. After correction, only one SNP at a known RLS risk locus, the *MEIS1* SNP rs113851554, showed a significant association in MetaGER (*p* = 5.73e-5), while it narrowly missed the corrected significance threshold in MetaALL (*p* = 3.51e-4) and was not significant in GRK (*p* = 0.942). Notably, it is the SNP, which also showed the lowest *p*-value in the latest large meta-analysis on idiopathic RLS [8] (*p* = 1.20e1105). Summary statistics for the subset of rls risk loci SNPs for MetaGER, GRK and MetaALL can be found in Tables S1–S3. Furthermore, two loci on chromosomes 7 and 22 strive for genome-wide significance, but may not reach it due to limited sample size in MetaALL (Figure 1a). The lead SNP of the locus on chromosome 7 is rs36127502 (*p* = 1.37e-7, OR = 1.77[1.43, 2.20], EAF = 0.22), which is intronic in *ABCA13* and an eQTL for *UPP1* in cerebellum when using GTEx V10 https://www.gtexportal.org/home/snp/rs36127502. The lead SNP of the locus on chromosome 22 is rs5769388 (*p* = 1.92e-7, OR = 1.73[1.41, 2.12], EAF = 0.55), which is 10 kb downstream of *NHIP*. None of these two SNPs/loci has been reported as genome-wide significant in GWAS on idiopathic RLS according to the GWAS catalog (https://www.ebi.ac.uk/gwas/home), and our latest GWAS meta-analysis on idiopathic RLS (rs36127502: *p* = 0.478, rs5769388: *p* = 0.635, [8]). Locus zoom plots for the two lead SNPs can be found in the Figures S1 and S2. Further validation in independent datasets is required to evaluate the reliability of these putative associations.

**Table 2:** Nominal significant SNPs in MetaGER filtered for 164 idiopathic RLS risk loci identified in [8]

rsid	chr	BP	EA	OA	EAF	OR (95% CI)	*p*-value	Direction
rs113851554	2	66 523 432	T	G	0.07	2.52[1.61, 3.96]	5.73e-05	++
rs4708178	6	75 311 622	A	G	0.31	0.69[0.54, 0.89]	.005	– –
rs306960	8	140 995 146	T	C	0.41	0.71[0.55, 0.90]	.005	– –
rs10947738	6	38 501 411	A	C	0.70	1.42[1.10, 1.82]	.007	++
rs6121424	20	61 527 190	T	C	0.07	1.94[1.18, 3.22]	.010	++
rs851663	7	147 814 797	T	G	0.33	0.74[0.58, 0.94]	.013	– –
rs12218808	10	14 278 991	T	G	0.78	0.74[0.57, 0.96]	.022	– –
rs4746361	10	76 093 226	T	C	0.44	1.30[1.03, 1.63]	.025	++
rs7099994	10	71 155 897	A	G	0.84	0.67[0.47, 0.95]	.026	– –
rs607289	15	47 783 356	A	G	0.39	0.78[0.62, 0.98]	.033	– –
rs4596705	9	9 214 833	T	G	0.41	0.79[0.63, 0.99]	.039	– –
rs2748809	14	99 268 896	T	C	0.60	1.29[1.00, 1.66]	.046	++

EA denotes the effect allele, OA denotes the other allele, chr stands for the chromosome, and BP is the base-pair position based on GRCh38/hg38. EAF is the allele frequency of the effect allele as provided by METAL. OR denotes the meta-analysis odds ratio estimate for EA. Direction gives the effect direction in GER1 and GER2, in that order.

**Table 3:** Nominal significant SNPs in GRK summary statistics filtered for 164 idiopathic RLS risk loci identified in [8]

rsid	chr	BP	EA	OA	EAF	OR (95% CI)	*p*-value
rs7176440	15	36 594 039	A	T	0.62	1.51[1.09, 2.11]	.013
rs62562210	9	117 077 250	A	G	0.14	0.56[0.34, 0.93]	.020
rs6438436	3	118 103 302	T	C	0.81	1.70[1.06, 2.75]	.023
rs10193366	2	4 654 893	A	G	0.23	0.63[0.42, 0.95]	.023
rs2914339	5	171 320 267	C	A	0.65	1.56[1.04, 2.34]	.028
rs9369006	6	37 529 102	G	C	0.20	0.60[0.37, 0.96]	.028
rs1326611	6	51 724 291	T	A	0.38	0.71[0.50, 0.99]	.040
rs681241	1	208 127 146	G	T	0.12	1.61[1.01, 2.57]	.049

EA denotes the effect allele, OA denotes the other allele, chr stands for the chromosome, and BP is the base-pair position based on GRCh38/hg38. EAF is the effect allele frequency. OR denotes the odds ratio estimate for EA.

**Table 4:** Nominal significant SNPs in MetaAll filtered for 164 idiopathic RLS risk loci identified in [8]

rsid	chr	BP	EA	OA	EAF	OR (95% CI)	*p*-value	Direction
rs113851554	2	66 523 432	T	G	0.06	2.06[1.39, 3.06]	3.51e-04	+++
rs10947738	6	38 501 411	A	C	0.69	1.39[1.13, 1.72]	.002	+++
rs4746361	10	76 093 226	T	C	0.44	1.28[1.06, 1.55]	.012	+++
rs306960	8	140 995 146	T	C	0.40	0.78[0.64, 0.95]	.014	– – –
rs72718216	14	68 989 056	C	G	0.90	0.70[0.53, 0.93]	.014	– – –
rs4708178	6	75 311 622	A	G	0.31	0.78[0.63, 0.96]	.017	– – –
rs6121424	20	61 527 190	T	C	0.08	1.57[1.07, 2.28]	.020	+++
rs10193366	2	4 654 893	A	G	0.22	0.76[0.61, 0.96]	.022	– – –
rs2748809	14	99 268 896	T	C	0.61	1.26[1.03, 1.54]	.027	+++
rs62562210	9	117 077 250	A	G	0.13	0.74[0.56, 0.98]	.035	– – –
rs851663	7	147 814 797	T	G	0.34	0.81[0.67, 0.99]	.035	– – –
rs4838313	9	126 030 679	A	G	0.31	0.80[0.65, 0.99]	.036	– – –
rs11793109	9	7 266 982	A	G	0.81	0.78[0.62, 0.99]	.039	+– –
rs73402082	11	8 405 830	T	G	0.84	1.32[1.01, 1.73]	.042	+++

EA denotes the effect allele, OA denotes the other allele, chr stands for the chromosome, and bp is the base-pair position based on GRCh38/hg38. EAF is the allele frequency of the effect allele as provided by METAL. OR denotes the meta-analysis odds ratio estimate for EA. Direction gives the effect direction in GER1, GER2, and GRK, in that order.

### RLS PRS results

**Figure 2:**
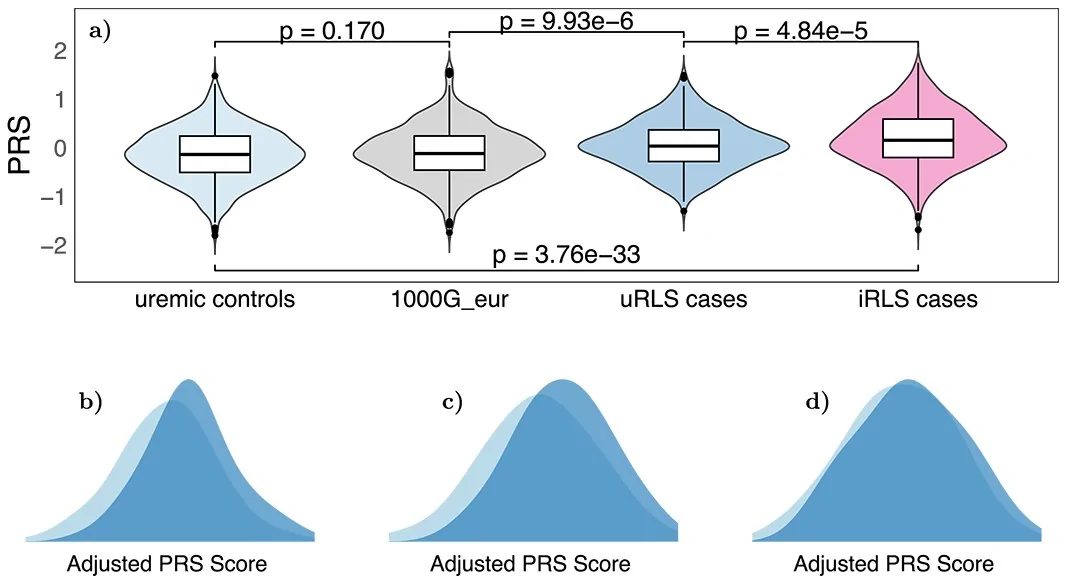
(a) Adjusted RLS PRS box plots for groups uremic controls, uremic RLS cases, European 1000G samples, idiopathic RLS cases. The groups are ordered by their median RLS PRS, increasing from left to right. The PRS was adjusted using PCs based on all groups combined and HapMap SNPs. (b)–(d) Estimated densities of adjusted RLS PRS distributions per uremic study population GER1, GER2, and GRK, respectively. Dark blue densities represent cases and light blue densities represent controls. For density estimation R’s ggplot2 command geom density with the parameter adjust = 1.5 was used.

Of the 216 SNPs in the RLS PRS defined in [8], 183 could be retrieved in the data sets GER1, GER2, GRK, iRLS cases, and 1000G eur with sufficient info score (R2). After applying the proxy procedure described in the methods section, 209 SNPs were included in an adapted PRS that was calculated in each data set, reducing SNP missingness from about 15% to 3% . For the Analysis of mean RLS PRS across the groups uremic controls, uRLS cases, 1000G eur and iRLS cases, a one-way Welch ANOVA showed that there was a significant difference of the means between at least two groups (F(3,1163) = 58.13, *p <* 5.14e-35). The results of pairwise Welchs’ *t*-tests can be found in Table 5, with box and violin plots in Figure 2a. The sensitivity analysis of mean RLS PRS in uRLS cases versus uremic controls as described in the methods section yielded the following results: Across the three uremic datasets, age- and sex-adjusted linear regression models showed the following associations of RLS status with RLS PRS: GER1 (*OR* = 1.31, 95% CI [1.19, 1.45], *p* = 1.66e-07), GER2 (*OR* = 1.26, 95% CI [1.09, 1.44], *p* = .001) and GRK (*OR* = 1.11, 95% CI [1.00, 1.25], *p* = .061). Random-effect meta-analysis (REML estimator, k = 3) combining these results showed a significant overall association (*OR* = 1.23, 95% CI [1.11, 1.36], *p* = 8.92e-05). Moderate between-study heterogeneity was observed (*τ*^2^ = 4.53e-03, *I*^2^ = 56.44%, Q(2) = 4.67, *p* = 0.1). The estimated RLS PRS distribution grouped by RLS status of the uremic datasets can be found in Figure 2b to d. The full summary statistics for linear regression models and pairwise tests can be found in Note S1. Rerunning the analysis without the ESRD individuals with known positive RLS family history results showed the same significant group differences (Table S5; Note S2). Regarding the comparison of mean adjusted RLS PRS between uremic RLS cases and the subset of 306 iRLS cases with available age information, a linear regression model with adjusted PRS as outcome and RLS type (uremic or idiopathic), age and sex as covariates found age and sex not to be significantly associated with adjusted PRS (age: *p* = .579, sex: *p* = .590), while the association with RLS type was highly significant (*p* = 2.66e-6, *OR* = 0.81, 95% CI [0.75, 0.89]).

**Table 5:** Welch’s *t-*test results for RLS PRS

Test	Mean difference (95%CI)	*p*-value
uRLS cases vs iRLS cases	−0.13 [-0.20, -0.07]	4.84e-05
uremic controls vs iRLS cases	−0.33 [-0.39, -0.28]	3.76e-33
uRLS cases vs 1000G eur	0.16 [0.09, 0.23]	9.93e-06
uremic controls vs 1000G eur	−0.04 [-0.10, 0.02]	.170
uRLS cases vs uremic controls	0.20 [0.14, 0.26]	9.47e-10
iRLS cases vs 1000G eur	0.29 [0.23, 0.35]	3.03e-20

Column one indicates the tested pair, column two shows the mean RLS PRS difference between the tested groups with the 95% confidence interval, and column three gives the *p*-value from Welch’s *t-*test.

### PRS of comorbidities

PRS of nine RLS comorbid traits (see Methods) were analyzed across the groups uremic controls, uRLS cases, 1000G eur, and iRLS cases. One-way Welch ANOVA of PRS of each of the traits found a significant result at the significance level of *α* = 0*.*05 for the traits T2D (F(3,1135.54) = 2.97, *p* = .031) and hypertension (F(3,1131.30) = 2.78, *p* = .040). Detailed summary statistics of the subsequent pairwise Welchs’ *t*-tests can be found in Notes S3–S11 and here we present only some relationships of groups. Mean T2D PRS was increased compared to 1000G eur for the groups uRLS cases (*Δmean* = 0*.*06, 95% CI [-0.02, 0.15], *p* = .118), iRLS cases (*Δmean* = 0*.*09, 95% CI [0.02, 0.15], *p* = .008) and uremic controls (*Δmean* = 0*.*09, 95% CI [0.03, 0.16], *p* = .006). Mean hypertension PRS was higher in uRLS cases than in the groups iRLS cases (*Δmean* = 0*.*03, 95% CI [4.40e-03, 0.06], *p* = .024), uremic controls (*Δmean* = 0*.*03, 95% CI [4.70e-03, 0.06], *p* = .022) and 1000G eur (*Δmean* = 0*.*04, 95% CI [0.01, 0.08], *p* = .005). An overview of the results of all pairwise tests for T2D PRS and hypertension PRS is provided in Table S6.

### Exploratory analysis of GER2 phenotype data

The patients underwent either hemodialysis (HD), hemodiafiltration (HDF), or in one case hemofiltration (HF). In the statistical analysis the HF patient was not included due to concerns regarding statistical stability. Of the remaining 255 individuals, 79 were affected by RLS. Table 6 indicates the distribution of their clinical variables. At the nominal significance level of 0.05, only regular smoking was significantly associated with RLS, showing an odds ratio (OR) of 2.23(CI 95%[1.17,4.24]). For a combined model the variables dialysis dosage, Kt/V, calcium, parathormone, CRP, smoking status, and alcohol consumption were selected due to their *p*-values in the univariate analyses being below the threshold of 0.2 (see Methods). Dialysis vintage (OR = 1.00, *p* = .130) was not included because the data was missing in more than 25% of study participants. Thus, the combined logistic regression model comprised 227 individuals with complete data for 7 variables (Table 7), of whom 71 were RLS positive. Nagelkerke’s pseudo R2 of the combined model amounted to 0.11. The Hosmer and Lemeshow goodness of fit test did not indicate an inadequacy of the model (*p* = .524). At the significance level of 0.05 the variables parathormone (*p* = .045) and smoking status (*p* = .023) showed significant association to the RLS status. While parathormone level had a rather small effect size with an OR of 1.01(CI 95% [1.00,1.02]), regular smoking showed a moderate effect on having RLS with an OR of 2.21(CI 95% [1.12,4.37]). All results can be found in Table 7.

**Table 6:** Univariate association tests with RLS status

Variable	Total	Control	Case	OR (95% CI)	*p*-value	N (%)
Sex (m)	180	125	55	0.94 [0.52, 1.67]	.820	255 (100)
Age	67 [54 - 77]	67 [54 - 77]	66 [54 - 76]	0.99 [0.98, 1.01]	.575	255 (100)
BMI	25.43 [22.71 - 28.44]	25.35 [22.65 - 28.48]	25.53 [22.8 - 28.38]	1.01 [0.97, 1.06]	.676	255 (100)
Dialysis dosage [h/w]	13.5 [12.5 - 13.5]	13.5 [12.5 - 13.5]	13.5 [12.5 - 14.25]	1.06 [0.97, 1.15]	.200	248 (97.25)
Dialysis method (HDF)	78	52	26	1.17 [0.66, 2.07]	.590	255 (100)
Dialysis vintage [months]	51 [27 - 94.5]	47.5 [26.25 - 88.75]	64 [29 - 120]	1.00 [1.00, 1.01]	.130	187 (73.33)
Ferritin [ng/ml]	622.8 [396.5 - 907.68]	604.8 [376 - 926.7]	651.1 [464.6 - 848.8]	1.00 [1.00, 1.00]	.954	252 (98.82)
Transferrin saturation [%]	25 [19 - 33]	24 [18 - 32]	26 [20 - 33.5]	1.01 [0.99, 1.03]	.332	255 (100)
Hemoglobin [g/dL]	11.6 [10.95 - 12.4]	11.6 [10.9 - 12.45]	11.6 [11.15 - 12.4]	0.97 [0.78, 1.20]	.779	255 (100)
Hematocrit [%]	34.8 [32.6 - 38]	35 [32.4 - 38]	34.4 [32.95 - 37.55]	0.99 [0.93, 1.05]	.728	255 (100)
URR	73.81 [69.2 - 77.5]	73.32 [68.57 - 76.64]	74.43 [70.03 - 78.04]	1.02 [0.98, 1.06]	.260	255 (100)
KtV	1.57 [1.38 - 1.77]	1.54 [1.36 - 1.74]	1.61 [1.42 - 1.81]	1.90 [0.95, 3.80]	.070	255 (100)
Cholesterol [mg/dl]	175 [144 - 208]	169 [142.75 - 206.25]	185 [152 - 209]	1.00 [1.00, 1.01]	.220	255 (100)
Calcium [mmol/l]	2.23 [2.13 - 2.31]	2.22 [2.11 - 2.3]	2.23 [2.15 - 2.32]	3.94 [0.81, 19.08]	.089	255 (100)
Phosphate [mg/dl]	5.18 [4.34 - 6.19]	5.1 [4.17 - 6.1]	5.27 [4.65 - 6.44]	1.10 [0.93, 1.29]	.260	255 (100)
Parathormone [pmol/l]	29.17 [13.95 - 48.1]	27.35 [12.41 - 48.46]	32.85 [15.65 - 47.38]	1.01 [1.00, 1.02]	.091	254 (99.61)
CRP [mg/l]	4 [2.9 - 9]	4 [2.46 - 7.78]	4 [3 - 12]	1.02 [0.99, 1.04]	.151	249 (97.65)
Leucocytes [giga/l]	6.7 [5.3 - 8]	6.65 [5.3 - 7.9]	6.7 [5.3 - 8.1]	1.00 [0.87, 1.14]	.996	255 (100)
HbA1c [%]	5.6 [5.3 - 6.5]	5.6 [5.3 - 6.5]	5.7 [5.4 - 6.5]	1.06 [0.81, 1.39]	.677	182 (71.37)
TSH [mU/l]	1.19 [0.73 - 1.79]	1.18 [0.7 - 1.79]	1.19 [0.81 - 1.76]	1.03 [0.83, 1.29]	.774	226 (88.63)
Diabetes (+)	102	70	32	1.03 [0.60, 1.77]	.912	255 (100)
Smoker (+)	48	26	22	2.23 [1.17, 4.24]	.015	255 (100)
Alcohol consumption (+)	73	56	17	0.59 [0.32, 1.10]	.095	255 (100)

Column two to four represent groupings of the data: The second column contains information on all samples for each variable, while column three and four separate the data according to RLS status for each variable. These columns contain the median and IQR in the case of continuous variables and the number of patients of the indicated group in the variable column in the case of dichotomous variables. Columns five and six contain the results of the univariate logistic regression models for each variable with RLS status as the response variable. The OR are provided with a 95% confidence interval. The last column contains the number of patients for which data on each variable was available and in brackets the percentage of non missing data can be found.

**Table 7:** Logistic regression model with RLS status as response variable

Variable	OR (95% CI)	*p*-Value
Dialysis dosage [h/w]	1.05 [0.96, 1.15]	.273
KtV	1.96 [0.89, 4.30]	.093
Calcium [mmol/l]	3.19 [0.58, 17.45]	.181
Parathormone [pmol/l]	1.01 [1.00, 1.02]	.045
CRP [mg/l]	1.02 [0.99, 1.04]	.168
Smoker (+)	2.21 [1.12, 4.37]	.023
Alcohol (+)	0.65 [0.34, 1.26]	.202

The second and third column represent the resulting OR with the 95% CI and the *p*-values of the combined logistic regression model with dialysis dosage, Kt/V, Calcium, parathormone, CRP, Smoking status and alcohol consumption as covariates.

## Discussion

This is the first genome-wide association analysis of RLS in ESRD and provides evidence that uremic RLS shares part of its genetic background with idiopathic RLS, particularly through the MEIS1 locus and the elevated idiopathic RLS PRS in uRLS cases. We performed GWAS of uremic RLS in three ESRD populations, one from Greece, two from Germany. Two of these cohorts, GER1 and GRK, have been examined in a previous study already, but only with regard to a small selection of SNPs at the gene loci of *MEIS1, BTBD9, MAP2K5, SKOR1*, and *PTPRD* [6]. At the nominal significance level (*p* = 0.05) the previous study found three significant associations at the *MEIS1* and *BTBD9* loci in the German study population. These SNPs remained significant in MetaGER, the meta-analysis of the two German GWAS datasets in the current study. For the Greek population the previous study reported a significant association (*p* = 0.021) with a SNP of *MAP2K5/SKOR1*, which in our analysis only showed a trend towards significance (*p* = 0.085). This discrepancy may be due to differences in the final analyzed sample size after genotype quality control in the GRK study population. When comparing the results of an analysis combining German and Greek individuals in [6] with MetaALL, the meta-analysis of all three GWAS datasets in the present study, one of the four SNPs at the *MAP2K5/SKOR1* locus that had been significant in [6] remained significant in our study. The only significant *BTBD9* SNP in [6] also remained significant in MetaALL. Detailed summary statistics of the SNPs of the discussed loci can be found in Table S4.

According to our knowledge, only one other study addressed the genetics of uremic RLS. That study was conducted in a cohort of Taiwanese dialysis patients [7] and also analyzed only a selection of SNPs (at the *MEIS1, BTBD9, MAP2K5/SKOR1, PTPRD, TOX3/bc034767* loci and at the intergenic region of chromosome 2p14), identifying a modest but significant association with RLS at the *PTPRD* locus (*p* = .03) [7]. Our current analysis could not replicate this association (*p* = .277, MetaALL), possibly due to the much lower MAF in the European ancestry (0.1 versus 0.4). Detailed summary statistics of the SNPs of the discussed loci can be found in Table S4.

The present study has extended these previous studies on uremic RLS by performing the first GWAS on RLS in ESRD patients and by comparing genetic risks for RLS across groups of idiopathic RLS cases, European 1000G individuals as a proxy for the general population, patients with ESRD but without RLS, and uremic RLS cases.

Grouping the ESRD patients from three uremic datasets into uremic RLS cases and uremic controls, an existing PRS trained on idiopathic RLS cases and controls showed a significantly increased mean in the uremic RLS cases versus the uremic controls. The same was true, when comparing the RLS PRS mean of uremic RLS cases and the European 1000G samples. These findings suggest that idiopathic RLS and uremic RLS share a considerable amount of genetic architecture. Another observation we made is that the mean PRS of idiopathic RLS cases was significantly higher than the mean PRS of uremic RLS cases. Under a model of additive factors causing RLS liability, individuals with ESRD need less genetic RLS risk to develop RLS due to the additional factors provided by ESRD. By the same logic [19], the trend towards lower mean PRS of uremic controls compared to the presumably healthy European 1000G samples could be explained by these ESRD patients without RLS having nonrisk variants at the genetic risk loci that counteract the ESRD-related RLS risk.

We found that the known *MEIS1* risk locus for iRLS is also significantly associated with uremic RLS in the German uremic populations when correcting for multiple testing based on all known RLS risk loci. In contrast, no significant association was observed in the Greek study population. When considering the results from the three GWAS separately, including the effect estimate on the log odds scale, effect allele frequency (EAF), standard error (SE), and the imputation *R*^2^ score, we found consistent significant associations in the two German study populations GER1 (*p* = .002, OR = 2.30[1.36, 3.89], EAF = 0.06, *R*^2^ = 0.91) and GER2 (*p* = .009, OR = 3.26[1.35, 7.85], EAF = 0.07, *R*^2^ = 0.76). In GRK, however, the effect estimate was close to zero and non-significant (*p* = .942, OR = 1.03[0.45, 2.37], EAF = 0.05, *R*^2^ = 0.70). This pattern likely accounts for the weaker association observed in MetaALL compared with MetaGER.

In the genome-wide meta-analysis MetaALL we found two loci with suggestive evidence of association (rs36127502: *p* = 1.37e-7, rs5769388: *p* = 1.92e-7) that have not been reported as risk loci for idiopathic RLS so far (rs36127502: *p* = .478, rs5769388: *p* = .635, [8]), which suggests a connection between uremia and these genetic variants. This could indicate that uremic RLS, despite sharing genetic architecture with idiopathic RLS, has some additional independent genetic factors contributing to the symptoms. Larger independent cohorts of ESRD patients with harmonized RLS phenotyping will be required to determine whether these signals reflect true uRLS associated genetic risk factors.

T2D and hypertension are known comorbidities of RLS and are also major risk factors for chronic kidney disease, each showing complex bidirectional relationships with chronic kidney disease [20, 21]. In our exploratory analysis, mean T2D PRS showed a trend towards an increase in patients with uremic RLS compared to European individuals from the 1000G sample who were not selected for RLS or ESRD. In patients with RLS only and in patients with ESRD only, mean T2D PRS was significantly increased compared with the same reference group. These findings may point to pleiotropic effects or shared genetic architecture of T2D, ESRD, and RLS, but should be treated as hypothesis-generating. Furthermore, we saw increased hypertension PRS in patients with uremic RLS compared to patients with ESRD but without RLS, European individuals in the 1000G sample and patients with idiopathic RLS. These exploratory results raise the possibility that hypertension genetics may contribute specifically to the cooccurrence of ESRD and RLS compared to each disease on its own. Follow-up analyses are needed to differentiate between mediation effects between the traits and true pleiotropic effects.

Various factors have been proposed as specific drivers of uremic RLS, including iron deficiency-related dopaminergic dysfunction similar to the role iron deficiency is supposed to have in idiopathic RLS, peripheral neuropathy, calcium/phosphate imbalances, and general uremic toxicity due to chronic kidney disease [22, 23]. Therefore, we examined laboratory and behavioral data available in GER2 for association with RLS. In a multivariate logistic regression model we identified a significant but small increase in risk of RLS if dialysis patients had increased parathormone levels, and a significant moderate increase of RLS risk if dialysis patients were regular smokers. Other studies examining risk factors for RLS in dialysis patients found inconsistent results: A study on an Iranian cohort reported a significant association with smoking [24] which was not confirmed in a Scottish study, however [25]. With regard to parathormone levels the findings are even more controversial: While a study on 579 Greek hemodialysis patients found a significant association between increased parathormone levels and a positive RLS status [26], parathormone levels were found to be significantly decreased in RLS in a study on 136 German hemodialysis patients [27]. Other studies with small sample sizes did not find any significant associations [28, 29]. Assuming that there is a positive association between parathormone levels and RLS status in ESRD patients, it remains to be examined if this association results from a causal relation or if it is an epiphenomenon. Secondary hyperparathyreoidism is frequent in ESRD/dialysis due to a lack of renal vitamin D activation, hyperphosphatemia, and hypocalcemia. However, the levels of phosphate and calcium were not associated with RLS in the present study. A meta-analysis on risk factors for RLS in dialysis patients previously revealed a significant association between lower hemoglobin levels in Caucasian RLS [30], which could not be replicated by the present study; however, the direction of the association was consistent.

The present study has some limitations. First, statistical power was limited by sample size. The absence of genome-wide significant findings should not be interpreted as absence of genetic contribution. Rather, the sample size provided power mainly to detect moderate-to-large effects, whereas most common variants for complex neurological traits have smaller effects. Second, detailed phenotype information was only available for one of the three datasets. Another limitation is that there was no information on the temporal sequence of RLS and renal disease. Given the RLS prevalence of 5.9% in the European population, a minority of idiopathic RLS cases may have been part of the analysis. However, when using familial RLS as a proxy for idiopathic RLS and excluding individuals with known family history, the PRS results remained unchanged. Nevertheless, due to the heterogeneity of ESRD patients, larger study populations with deeper phenotyping will be required to investigate phenotype associations and genetic effects more robustly. Finally, the findings are mostly European-ancestry based and may not generalize to other populations.

In summary, the present study suggests a shared genetic architecture of uremic RLS and idiopathic RLS while also pointing to potential genetic risk factors specific to uremic RLS. The lack of association signals in the MEIS1 locus in the Greek study population calls for a deeper investigation of the role of MEIS1 in RLS of this population. Our findings shed light on the genetic background of uremic RLS and provide a basis for further investigation into the causes of the increased prevalence of RLS in ESRD. At this stage, the findings do not support genetic testing or PRS-based clinical prediction of uRLS in ESRD patients. Their main value is pathophysiological, by suggesting overlap between idiopathic and uremic RLS and identifying candidate signals for replication.

## Supplementary Material

Supplementary_material_zpag095

## Data Availability

The GWAS summary statistics for GER1, GER2, and GRK and code used for statistical analyses are available at Zenodo (doi:10.5281/zenodo.21234712).
