# Supplementary material for: Genome-wide association study of restless legs syndrome in end-stage renal disease
Source: Sleep Adv. 2026 Aug 29;7(3):zpag095. doi: 10.1093/sleepadvances/zpag095 (PMC13589853; doi:10.1093/sleepadvances/zpag095)
Supplement: Supplementary file 1 [file supplementary_material_zpag095.zip › supplement_notes_GWAS_of_RLS_in_ESRD_by_Risse_et_al_zpag095.docx]

Genome-wide association study of restless legs syndrome in end-stage renal disease

Noah Risse^1,2,^*, Philip Harrer^1,2^, Giorgos K Sakkas^3,4^, Ioannis Stefanidis^5^, Nathalie Schandra^1,2^, Ambra Stefani^6^, Magdalena Wildt^6^, Yves Dauvilliers^7^, Georgios M Hadjigeorgiou^8^, Chen Zhao^1,2^, Juliane Winkelmann^1,2,9,10^, Konrad Oexle^1,2,11,#^, Barbara Schormair^1,2,^*^,#^

*corresponding authors

#contributed equally

^1^Institute of Neurogenomics, Helmholtz Zentrum München, German Research Center for Environmental Health, Neuherberg, Germany

^2^Institute of Human Genetics, TUM School of Medicine and Health, Technical University of Munich, Munich, Germany

^3^Department of Physical Education and Sport Science, University of Thessaly, Thessaly, Trikala, 42100, Greece

^4^Department of Life Sciences, School of Life and Health Sciences, University of Nicosia, Nicosia, 2417, Cyprus

^5^Department of Nephrology, School of Medicine, University of Thessaly, Larisa, Greece

^6^Department of Neurology, Medical University of Innsbruck, Innsbruck, Austria

^7^Sleep–Wake Disorders Centre, Department of Neurology, Gui de Chauliac Hospital, Montpellier, France

^8^Department of Neurology, Nicosia General Hospital Medical School, University of Cyprus, Nicosia, Cyprus

^9^Munich Cluster for Systems Neurology (SyNergy), Munich, Germany

^10^German Center for Mental Health (DZPG), partner site Munich–Augsburg, Munich–Augsburg, Germany

^11^Neurogenetic Systems Analysis Group, Institute of Neurogenomics, Helmholtz Zentrum München, German Research Center for Environmental Health, Neuherberg, Germany

**Corresponding authors:**

Noah Risse

Ingolstädter Landstraße 1, 85764 Neuherberg, Germany, noah.risse@helmholtz-munich.de

Barbara Schormair

Ingolstädter Landstraße 1, 85764 Neuherberg, Germany, barbara.schormair@helmholtz-munich.de

Supplementary Material

## Contents

[**Supplementary Figures**	**3**](#_Toc282952)

[**Supplementary Note 1: RLS PRS**	**5**](#_Toc282953)

[**Supplementary Note 2: RLS PRS (without familial cases)**	**10**](#_Toc282954)

[**Supplementary Note 3: Type 2 diabetes PRS**	**15**](#_Toc282955)

[**Supplementary Note 4: Hypertension PRS**	**20**](#_Toc282956)

[**Supplementary Note 5: Depression PRS**	**25**](#_Toc282957)

[**Supplementary Note 6: Low density lipoprotein measurement PRS**	**27**](#_Toc282958)

[**Supplementary Note 7: Ischemic stroke PRS**	**29**](#_Toc282959)

[**Supplementary Note 8: Heart failure PRS**	**31**](#_Toc282960)

[**Supplementary Note 9: Coronary artery disease PRS**	**33**](#_Toc282961)

[**Supplementary Note 10: Anxiety PRS**	**35**](#_Toc282962)

[**Supplementary Note 11: Abdominal aortic aneurism PRS**	**37**](#_Toc282963)

# Supplementary Figures

Supplementary Figure 1: Locus zoom plot of rs36127502 using a 500kb window. LD is based on a European 1000g reference panel.

Supplementary Figure 2: Locus zoom plot of rs5769388 using a 500kb window. LD is based on a European 1000g reference panel. The Ensembl ID of AL078622.1 is ENSG00000286381 and is called the NHIP gene.

Supplementary Figure 3: Multidimensional scaling (MDS) plots of the first two components for the three uremic GWAS datasets projected onto individuals from the 1000G project. Panels A–C show GER1, GER2 and GRK respectively, after basic quality control steps but before excluding individuals that deviate more than 3 SD from the mean of any of the first 5 MDS components calculated within dataset without considering 1000G individuals. Panels D–F show the corresponding datasets after outlier exclusion.

Supplementary Figure 4: Panels A–C show scree plots of the eigenvalues for the first ten PCs calculated for GER1, GER2 and GRK, respectively.

# Supplementary Note 1: RLS PRS

Table N1.1: PRS information for RLS PRS

| Metric | Value |
| --- | --- |
| PRS | RLS |
| PGS catalog link | No link available |
| Available PRS SNPs | 209 |
| Unavailable PRS SNPs | 7 |
| SNP missingness | 3.24% |

Table N1.2: Shapiro–Wilk test results to assess normality of RLS PRS in the study groups. For each group, the Shapiro–Wilk test statistic W and corresponding P-value are reported.

| Group | W | P-value |
| --- | --- | --- |
| 1000G_eur | 1.00 | 0.181 |
| iRLS_cases | 1.00 | 0.332 |
| uremic_controls | 1.00 | 0.818 |
| uRLS_cases | 1.00 | 0.636 |

Figure N1.1: Group-wise Q-Q plots of RLS PRS. Within each group the PRS was scales prior to plotting. The plot allows visual assessment of deviations from normality.

**RLS PRS Score by group (in ascending order by median)**

Figure N1.2: RLS PRS Score by group in ascending order by median PRS

uremic_controls

1000

G_eur

uRLS_cases

iRLS_cases

−1.5

−0.5

0.5

1.0

1.5

PRS

Table N1.3: Welch one-way ANOVA comparing RLS PRS across groups. F statistic denotes Welch’s F statistic, Numerator df and Denominator df denote the numerator and denominator degrees of freedom and P-value denotes the corresponding p-value.

| Test | F statistic | Numerator df | Denominator df | P-value |
| --- | --- | --- | --- | --- |
| Welch one-way ANOVA | 58.13 | 3 | 1162.89 | 5.14e-35 |

Table N1.4: Results of pairwise Welch’s t-tests comparing mean RLS PRS between groups. Test pair denotes the two groups compared, Mean1 and Mean2 denote the group means for the first and second group in the test pair, Effect denotes the difference Mean1 - Mean2, t denotes the Welch’s t-statistic, 95% CI lower and 95% CI upper denote the bounds of the 95% confidence interval for the mean difference and P-value denotes the corresponding two-sided p-value.

| Test pair | Effect | Mean1 | Mean2 | t | 95% CI lower | 95% CI upper | P-value |
| --- | --- | --- | --- | --- | --- | --- | --- |
| uRLS_cases vs iRLS_cases | -0.13 | 0.05 | 0.18 | -4.09 | -0.20 | -0.07 | 4.84e-05 |
| uremic_controls vs iRLS_cases | -0.33 | -0.15 | 0.18 | -12.26 | -0.39 | -0.28 | 3.76e-33 |
| uRLS_cases vs 1000G_eur | 0.16 | 0.05 | -0.11 | 4.45 | 0.09 | 0.23 | 9.93e-06 |
| uremic_controls vs 1000G_eur | -0.04 | -0.15 | -0.11 | -1.37 | -0.10 | 0.02 | 0.170 |
| uRLS_cases vs uremic_controls | 0.20 | 0.05 | -0.15 | 6.20 | 0.14 | 0.26 | 9.47e-10 |
| iRLS_cases vs 1000G_eur | 0.29 | 0.18 | -0.11 | 9.40 | 0.23 | 0.35 | 3.03e-20 |

Table N1.5: Summary of RLS PRS values by RLS status for RLS in the GER1 dataset. N is the number of individuals in each group and Mean RLS PRS denotes the mean PRS in each group.

| Group | N | Mean RLS PRS |
| --- | --- | --- |
| RLS controls | 332 | -0.19 |
| RLS cases | 169 | 0.08 |

Table N1.6: Results of linear regression in the GER1 dataset with RLS PRS as outcome and RLS status, sex and age as predictors. 95% CI lower and 95% CI upper are the lower and upper bound of the 95% confidence interval for the regression coefficient Beta. SE is the estimated standard error of Beta, t denotes the t-statistic testing whether the estimated coefficient differs from zero and P-value denotes the corresponding two-sided p-value.

| Variable | Beta | SE | 95% CI lower | 95% CI upper | t | P-value |
| --- | --- | --- | --- | --- | --- | --- |
| (Intercept) | -0.41 | 0.14 | -0.69 | -0.14 | -2.91 | 0.004 |
| RLS_statuscase | 0.27 | 0.05 | 0.17 | 0.37 | 5.31 | 1.66e-07 |
| SEX | -0.02 | 0.05 | -0.12 | 0.07 | -0.47 | 0.639 |
| Age | 3.89e-03 | 1.92e-03 | 1.28e-04 | 7.66e-03 | 2.03 | 0.043 |

Table N1.7: Summary of RLS PRS values by RLS status for RLS in the GER2 dataset. N is the number of individuals in each group and Mean RLS PRS denotes the mean PRS in each group.

| Group | N | Mean RLS PRS |
| --- | --- | --- |
| RLS controls | 164 | -0.24 |
| RLS cases | 74 | -8.72e-03 |

Table N1.8: Results of linear regression in the GER2 dataset with RLS PRS as outcome and RLS status, sex and age as predictors. 95% CI lower and 95% CI upper are the lower and upper bound of the 95% confidence interval for the regression coefficient Beta. SE is the estimated standard error of Beta, t denotes the t-statistic testing whether the estimated coefficient differs from zero and P-value denotes the corresponding two-sided p-value.

| Variable | Beta | SE | 95% CI lower | 95% CI upper | t | P-value |
| --- | --- | --- | --- | --- | --- | --- |
| (Intercept) | -0.46 | 0.19 | -0.84 | -0.08 | -2.36 | 0.019 |
| RLS_statuscase | 0.23 | 0.07 | 0.09 | 0.37 | 3.22 | 0.001 |
| SEX | 0.04 | 0.07 | -0.10 | 0.19 | 0.57 | 0.568 |
| Age | 2.52e-03 | 2.28e-03 | -1.94e-03 | 6.98e-03 | 1.11 | 0.269 |

Table N1.9: Summary of RLS PRS values by RLS status for RLS in the GRK dataset. N is the number of individuals in each group and Mean RLS PRS denotes the mean PRS in each group.

| Group | N | Mean RLS PRS |
| --- | --- | --- |
| RLS controls | 319 | -0.06 |
| RLS cases | 118 | 0.05 |

Table N1.10: Results of linear regression in the GRK dataset with RLS PRS as outcome and RLS status, sex and age as predictors. 95% CI lower and 95% CI upper are the lower and upper bound of the 95% confidence interval for the regression coefficient Beta. SE is the estimated standard error of Beta, t denotes the t-statistic testing whether the estimated coefficient differs from zero and P-value denotes the corresponding two-sided p-value.

| Variable | Beta | SE | 95% CI lower | 95% CI upper | t | P-value |
| --- | --- | --- | --- | --- | --- | --- |
| (Intercept) | -0.05 | 0.14 | -0.33 | 0.23 | -0.32 | 0.746 |
| RLS_statuscase | 0.11 | 0.06 | -4.76e-03 | 0.22 | 1.88 | 0.061 |
| SEX | -1.78e-03 | 0.05 | -0.10 | 0.10 | -0.03 | 0.972 |
| Age | -2.14e-04 | 1.90e-03 | -3.94e-03 | 3.52e-03 | -0.11 | 0.911 |

control

case

−1.5

−1.0

−0.5

0.0

0.5

1.0

1.5

**GER1 dataset**

RLS_status

PRS_adj

control

case

−1.5

−1.0

−0.5

0.0

0.5

1.0

**GER2 dataset**

RLS_status

PRS_adj

control

case

−1.5

−1.0

−0.5

0.0

0.5

1.0

**GRK dataset**

RLS_status

PRS_adj

Figure N1.3: RLS PRS score by RLS status across the datasets GER1, GER2 and GRK.

Table N1.11: Random-effects meta-analysis of the association between RLS status and RLS PRS across the

GER1, GER2, and GRK datasets. The meta-analysis used the regression coefficients and standard errors for RLS status from dataset-specific linear regression models with RLS PRS as the outcome and RLS status, sex, and age as predictors. The pooled effect was estimated using restricted maximum likelihood (REML). SE denotes the standard error of the pooled estimate; 95% CI lower and 95% CI upper denote the bounds of the 95% confidence interval, z denotes the test statistic for the pooled effect, and p denotes the corresponding p-value. Cochran’s Q, Q df, and Q-test p describe the test for heterogeneity, tau^2^ denotes the estimated between-dataset variance, I^2^ denotes the percentage of total variability attributable to heterogeneity and H^2^ denotes the relative excess in variability due to heterogeneity.

| Section | Statistic | Value |
| --- | --- | --- |
| Pooled effect | Method | REML |
| Pooled effect | Estimate | 0.20 |
| Pooled effect | SE | 0.05 |
| Pooled effect | 95% CI lower | 0.10 |
| Pooled effect | 95% CI upper | 0.30 |
| Pooled effect | z | 3.92 |
| Pooled effect | p | 8.92e-05 |
| Heterogeneity | Cochran’s Q | 4.67 |
| Heterogeneity | Q df | 2 |
| Heterogeneity | Q-test p | 0.097 |
| Heterogeneity | tau^2^ | 4.53e-03 |
| Heterogeneity | I^2^ (%) | 56.44 |
| Heterogeneity | H^2^ | 2.30 |

# Supplementary Note 2: RLS PRS (without familial cases)

Table N2.1: PRS information for RLS PRS

| Metric | Value |
| --- | --- |
| PRS | RLS without familial cases |
| PGS catalog link | no link |
| Available PRS SNPs | 209 |
| Unavailable PRS SNPs | 7 |
| SNP missingness | 3.24% |

Table N2.2: Shapiro–Wilk test results to assess normality of RLS PRS in the study groups. Uremic groups are without individuals with known familial RLS. For each group, the Shapiro–Wilk test statistic W and corresponding P-value are reported.

| Group | W | P-value |
| --- | --- | --- |
| 1000G_eur | 1.00 | 0.181 |
| iRLS_cases | 1.00 | 0.332 |
| uremic_controls | 1.00 | 0.827 |
| uRLS_cases | 1.00 | 0.590 |

Figure N2.1: Group-wise Q-Q plots of RLS PRS. Within each group the PRS was scales prior to plotting. Uremic groups are without individuals with known familial RLS. The plot allows visual assessment of deviations from normality.

**RLS without familial cases PRS Score by group (in ascending order by median)**

Figure N2.2: RLS PRS Score by group in ascending order by median PRS. Uremic groups are without individuals with known familial RLS.

uremic_controls

1000

G_eur

uRLS_cases

iRLS_cases

−1.5

−0.5

0.5

1.0

1.5

PRS

Table N2.3: Welch one-way ANOVA comparing RLS PRS across groups. Uremic groups are without individuals with known familial RLS. F statistic denotes Welch’s F statistic, Numerator df and Denominator df denote the numerator and denominator degrees of freedom and P-value denotes the corresponding p-value.

| Test | F statistic | Numerator df | Denominator df | P-value |
| --- | --- | --- | --- | --- |
| Welch one-way ANOVA | 57.06 | 3 | 1109.91 | 2.63e-34 |

Table N2.4: Results of pairwise Welch’s t-tests comparing mean RLS PRS between groups. Uremic groups are without individuals with known familial RLS. Test pair denotes the two groups compared, Mean1 and Mean2 denote the group means for the first and second group in the test pair, Effect denotes the difference Mean1 - Mean2, t denotes the Welch’s t-statistic, 95% CI lower and 95% CI upper denote the bounds of the 95% confidence interval for the mean difference and P-value denotes the corresponding two-sided p-value.

| Test pair | Effect | Mean1 | Mean2 | t | 95% CI lower | 95% CI upper | P-value |
| --- | --- | --- | --- | --- | --- | --- | --- |
| uRLS_cases vs iRLS_cases | -0.16 | 0.03 | 0.18 | -4.75 | -0.22 | -0.09 | 2.46e-06 |
| uremic_controls vs iRLS_cases | -0.33 | -0.15 | 0.18 | -12.26 | -0.39 | -0.28 | 3.78e-33 |
| uRLS_cases vs 1000G_eur | 0.13 | 0.03 | -0.11 | 3.73 | 0.06 | 0.21 | 2.04e-04 |
| uremic_controls vs 1000G_eur | -0.04 | -0.15 | -0.11 | -1.39 | -0.10 | 0.02 | 0.165 |
| uRLS_cases vs uremic_controls | 0.18 | 0.03 | -0.15 | 5.39 | 0.11 | 0.24 | 9.55e-08 |
| iRLS_cases vs 1000G_eur | 0.29 | 0.18 | -0.11 | 9.40 | 0.23 | 0.35 | 3.03e-20 |

Table N2.5: Summary of RLS PRS values by RLS status in the GER1 dataset. Individuals with known familial RLS were removed. N is the number of individuals in each group and Mean RLS PRS denotes the mean PRS in each group.

| Group | N | Mean RLS PRS |
| --- | --- | --- |
| RLS controls | 332 | -0.19 |
| RLS cases | 151 | 0.06 |

Table N2.6: Results of linear regression in the GER1 dataset with RLS PRS as outcome and RLS status, sex and age as predictors. Individuals with known familial RLS were removed. 95% CI lower and 95% CI upper are the lower and upper bound of the 95% confidence interval for the regression coefficient Beta. SE is the estimated standard error of Beta, t denotes the t-statistic testing whether the estimated coefficient differs from zero and P-value denotes the corresponding two-sided p-value.

| Variable | Beta | SE | 95% CI lower | 95% CI upper | t | P-value |
| --- | --- | --- | --- | --- | --- | --- |
| (Intercept) | -0.39 | 0.15 | -0.68 | -0.11 | -2.69 | 0.007 |
| RLS_statuscase | 0.25 | 0.05 | 0.15 | 0.36 | 4.77 | 2.46e-06 |
| SEX | -0.04 | 0.05 | -0.14 | 0.06 | -0.80 | 0.425 |
| Age | 3.88e-03 | 1.94e-03 | 6.64e-05 | 7.69e-03 | 1.99 | 0.047 |

Table N2.7: Summary of RLS PRS values by RLS status in the GER2 dataset. Individuals with known familial RLS were removed. N is the number of individuals in each group and Mean RLS PRS denotes the mean PRS in each group.

| Group | N | Mean RLS PRS |
| --- | --- | --- |
| RLS controls | 161 | -0.24 |
| RLS cases | 66 | -0.05 |

Table N2.8: Results of linear regression in the GER2 dataset with RLS PRS as outcome and RLS status, sex and age as predictors. Individuals with known familial RLS were removed. 95% CI lower and 95% CI upper are the lower and upper bound of the 95% confidence interval for the regression coefficient Beta. SE is the estimated standard error of Beta, t denotes the t-statistic testing whether the estimated coefficient differs from zero and P-value denotes the corresponding two-sided p-value.

| Variable | Beta | SE | 95% CI lower | 95% CI upper | t | P-value |
| --- | --- | --- | --- | --- | --- | --- |
| (Intercept) | -0.43 | 0.20 | -0.82 | -0.04 | -2.18 | 0.030 |
| RLS_statuscase | 0.20 | 0.07 | 0.05 | 0.34 | 2.67 | 0.008 |
| SEX | 0.03 | 0.08 | -0.12 | 0.18 | 0.42 | 0.674 |
| Age | 2.23e-03 | 2.30e-03 | -2.28e-03 | 6.74e-03 | 0.97 | 0.333 |

Table N2.9: Summary of RLS PRS values in the GRK dataset. Individuals with known familial RLS were removed. N is the number of individuals in each group and Mean RLS PRS denotes the mean PRS in each group.

| Group | N | Mean RLS PRS |
| --- | --- | --- |
| RLS controls | 319 | -0.06 |
| RLS cases | 112 | 0.02 |

Table N2.10: Results of linear regression in the GRK dataset with RLS PRS as outcome and RLS status, sex and age as predictors. Individuals with known familial RLS were removed. 95% CI lower and 95% CI upper are the lower and upper bound of the 95% confidence interval for the regression coefficient Beta. SE is the estimated standard error of Beta, t denotes the t-statistic testing whether the estimated coefficient differs from zero and P-value denotes the corresponding two-sided p-value.

| Variable | Beta | SE | 95% CI lower | 95% CI upper | t | P-value |
| --- | --- | --- | --- | --- | --- | --- |
| (Intercept) | -0.03 | 0.14 | -0.31 | 0.25 | -0.18 | 0.855 |
| RLS_statuscase | 0.08 | 0.06 | -0.03 | 0.20 | 1.45 | 0.147 |
| SEX | -0.02 | 0.05 | -0.12 | 0.08 | -0.34 | 0.734 |
| Age | -1.88e-04 | 1.90e-03 | -3.91e-03 | 3.53e-03 | -0.10 | 0.921 |

control

case

−1.5

−1.0

−0.5

0.0

0.5

1.0

1.5

**GER1 dataset**

RLS_status

PRS_adj

control

case

−1.5

−1.0

−0.5

0.0

0.5

1.0

**GER2 dataset**

RLS_status

PRS_adj

control

case

−1.5

−1.0

−0.5

0.0

0.5

1.0

**GRK dataset**

RLS_status

PRS_adj

Figure N2.3: RLS PRS score by RLS status across the datasets GER1, GER2 and GRK. Individuals with known familial RLS were removed.

Table N2.11: Random-effects meta-analysis of the association between RLS status and RLS PRS across the GER1, GER2, and GRK datasets. Individuals with known familial RLS were removed. The meta-analysis used the regression coefficients and standard errors for RLS status from dataset-specific linear regression models with RLS without familial cases PRS as the outcome and RLS status, sex, and age as predictors. The pooled effect was estimated using restricted maximum likelihood (REML). SE denotes the standard error of the pooled estimate; 95% CI lower and 95% CI upper denote the bounds of the 95% confidence interval, z denotes the test statistic for the pooled effect, and p denotes the corresponding p-value. Cochran’s Q, Q df, and Q-test p describe the test for heterogeneity, tau^2^ denotes the estimated between-dataset variance, I^2^ denotes the percentage of total variability attributable to heterogeneity and H^2^ denotes the relative excess in variability due to heterogeneity.

| Section | Statistic | Value |
| --- | --- | --- |
| Pooled effect | Method | REML |
| Pooled effect | Estimate | 0.18 |
| Pooled effect | SE | 0.05 |
| Pooled effect | 95% CI lower | 0.07 |
| Pooled effect | 95% CI upper | 0.28 |
| Pooled effect | z | 3.38 |
| Pooled effect | p | 7.27e-04 |
| Heterogeneity | Cochran’s Q | 4.58 |
| Heterogeneity | Q df | 2 |
| Heterogeneity | Q-test p | 0.101 |
| Heterogeneity | tau^2^ | 4.64e-03 |
| Heterogeneity | I^2^ (%) | 55.62 |
| Heterogeneity | H^2^ | 2.25 |

# Supplementary Note 3: Type 2 diabetes PRS

Table N3.1: PRS information for Type 2 diabetes PRS

| Metric | Value |
| --- | --- |
| PRS | Type 2 diabetes |
| PGS catalog link | <https://www.pgscatalog.org/score/PGS000805> |
| Available PRS SNPs | 554 |
| Unavailable PRS SNPs | 25 |
| SNP missingness | 4.32% |

Table N3.2: Shapiro–Wilk test results to assess normality of Type 2 diabetes PRS in the study groups. For each group, the Shapiro–Wilk test statistic W and corresponding P-value are reported.

| Group | W | P-value |
| --- | --- | --- |
| 1000G_eur | 1.00 | 0.368 |
| iRLS_cases | 1.00 | 0.231 |
| uremic_controls | 1.00 | 0.218 |
| uRLS_cases | 1.00 | 0.663 |

Figure N3.1: Group-wise Q-Q plots of Type 2 diabetes PRS. Within each group the PRS was scales prior to plotting. The plot allows visual assessment of deviations from normality.

**Type 2 diabetes PRS Score by group (in ascending order by median)**

Figure N3.2: Type 2 diabetes PRS Score by group in ascending order by median PRS

1000

G_eur

uRLS_cases

iRLS_cases

uremic_controls

−2

−1

0

1

2

PRS

Table N3.3: Welch one-way ANOVA comparing Type 2 diabetes PRS across groups. F statistic denotes

Welch’s F statistic, Numerator df and Denominator df denote the numerator and denominator degrees of freedom and P-value denotes the corresponding p-value.

| Test | F statistic | Numerator df | Denominator df | P-value |
| --- | --- | --- | --- | --- |
| Welch one-way ANOVA | 2.97 | 3 | 1135.54 | 0.031 |

Table N3.4: Results of pairwise Welch’s t-tests comparing mean Type 2 diabetes PRS between groups. Test pair denotes the two groups compared, Mean1 and Mean2 denote the group means for the first and second group in the test pair, Effect denotes the difference Mean1 - Mean2, t denotes the Welch’s t-statistic, 95% CI lower and 95% CI upper denote the bounds of the 95% confidence interval for the mean difference and P-value denotes the corresponding two-sided p-value.

| Test pair | Effect | Mean1 | Mean2 | t | 95% CI lower | 95% CI upper | P-value |
| --- | --- | --- | --- | --- | --- | --- | --- |
| uRLS_cases vs iRLS_cases | -0.02 | -4.32e-03 | 0.02 | -0.63 | -0.10 | 0.05 | 0.529 |
| uremic_controls vs iRLS_cases | 4.72e-03 | 0.02 | 0.02 | 0.16 | -0.05 | 0.06 | 0.871 |
| uRLS_cases vs 1000G_eur | 0.06 | -4.32e-03 | -0.07 | 1.57 | -0.02 | 0.15 | 0.118 |
| uremic_controls vs 1000G_eur | 0.09 | 0.02 | -0.07 | 2.74 | 0.03 | 0.16 | 0.006 |
| uRLS_cases vs uremic_controls | -0.03 | -4.32e-03 | 0.02 | -0.74 | -0.10 | 0.05 | 0.457 |
| iRLS_cases vs 1000G_eur | 0.09 | 0.02 | -0.07 | 2.65 | 0.02 | 0.15 | 0.008 |

Table N3.5: Summary of Type 2 diabetes PRS values by RLS status for Type 2 diabetes in the GER1 dataset. N is the number of individuals in each group and Mean Type 2 diabetes PRS denotes the mean PRS in each group.

| Group | N | Mean Type 2 diabetes PRS |
| --- | --- | --- |
| RLS controls | 332 | 0.03 |
| RLS cases | 169 | -0.01 |

Table N3.6: Results of linear regression in the GER1 dataset with Type 2 diabetes PRS as outcome and RLS status, sex and age as predictors. 95% CI lower and 95% CI upper are the lower and upper bound of the 95% confidence interval for the regression coefficient Beta. SE is the estimated standard error of Beta, t denotes the t-statistic testing whether the estimated coefficient differs from zero and P-value denotes the corresponding two-sided p-value.

| Variable | Beta | SE | 95% CI lower | 95% CI upper | t | P-value |
| --- | --- | --- | --- | --- | --- | --- |
| (Intercept) | 0.28 | 0.15 | -0.02 | 0.58 | 1.86 | 0.064 |
| RLS_statuscase | -0.03 | 0.05 | -0.13 | 0.08 | -0.52 | 0.604 |
| SEX | -0.16 | 0.05 | -0.26 | -0.06 | -3.06 | 0.002 |
| Age | -5.27e-04 | 2.04e-03 | -4.53e-03 | 3.47e-03 | -0.26 | 0.796 |

Table N3.7: Summary of Type 2 diabetes PRS values by RLS status for Type 2 diabetes in the GER2 dataset. N is the number of individuals in each group and Mean Type 2 diabetes PRS denotes the mean PRS in each group.

| Group | N | Mean Type 2 diabetes PRS |
| --- | --- | --- |
| RLS controls | 164 | 0.04 |
| RLS cases | 74 | 0.10 |

Table N3.8: Results of linear regression in the GER2 dataset with Type 2 diabetes PRS as outcome and RLS status, sex and age as predictors. 95% CI lower and 95% CI upper are the lower and upper bound of the 95% confidence interval for the regression coefficient Beta. SE is the estimated standard error of Beta, t denotes the t-statistic testing whether the estimated coefficient differs from zero and P-value denotes the corresponding two-sided p-value.

| Variable | Beta | SE | 95% CI lower | 95% CI upper | t | P-value |
| --- | --- | --- | --- | --- | --- | --- |
| (Intercept) | -0.28 | 0.23 | -0.74 | 0.18 | -1.19 | 0.236 |
| RLS_statuscase | 0.06 | 0.09 | -0.11 | 0.23 | 0.66 | 0.507 |
| SEX | 1.29e-03 | 0.09 | -0.17 | 0.18 | 0.01 | 0.988 |
| Age | 4.89e-03 | 2.76e-03 | -5.20e-04 | 0.01 | 1.77 | 0.078 |

Table N3.9: Summary of Type 2 diabetes PRS values by RLS status for Type 2 diabetes in the GRK dataset. N is the number of individuals in each group and Mean Type 2 diabetes PRS denotes the mean PRS in each group.

| Group | N | Mean Type 2 diabetes PRS |
| --- | --- | --- |
| RLS controls | 319 | 0.01 |
| RLS cases | 118 | -0.06 |

Table N3.10: Results of linear regression in the GRK dataset with Type 2 diabetes PRS as outcome and RLS status, sex and age as predictors. 95% CI lower and 95% CI upper are the lower and upper bound of the 95% confidence interval for the regression coefficient Beta. SE is the estimated standard error of Beta, t denotes the t-statistic testing whether the estimated coefficient differs from zero and P-value denotes the corresponding two-sided p-value.

| Variable | Beta | SE | 95% CI lower | 95% CI upper | t | P-value |
| --- | --- | --- | --- | --- | --- | --- |
| (Intercept) | 0.08 | 0.17 | -0.25 | 0.40 | 0.46 | 0.648 |
| RLS_statuscase | -0.06 | 0.07 | -0.19 | 0.07 | -0.89 | 0.375 |
| SEX | -0.09 | 0.06 | -0.21 | 0.03 | -1.54 | 0.125 |
| Age | 9.49e-04 | 2.24e-03 | -3.43e-03 | 5.33e-03 | 0.42 | 0.671 |

control

case

−1.5

−1.0

−0.5

0.0

0.5

1.0

1.5

**GER1 dataset**

RLS_status

PRS_adj

control

case

−1.5

−1.0

−0.5

0.0

0.5

1.0

1.5

**GER2 dataset**

RLS_status

PRS_adj

control

case

−1.5

−1.0

−0.5

0.0

0.5

1.0

1.5

2.0

**GRK dataset**

RLS_status

PRS_adj

Figure N3.3: Type 2 diabetes PRS score by RLS status across the datasets GER1, GER2 and GRK.

Table N3.11: Random-effects meta-analysis of the association between RLS status and Type 2 diabetes PRS across the GER1, GER2, and GRK datasets. The meta-analysis used the regression coefficients and standard errors for RLS status from dataset-specific linear regression models with Type 2 diabetes PRS as the outcome and RLS status, sex, and age as predictors. The pooled effect was estimated using restricted maximum likelihood (REML). SE denotes the standard error of the pooled estimate; 95% CI lower and 95% CI upper denote the bounds of the 95% confidence interval, z denotes the test statistic for the pooled effect, and p denotes the corresponding p-value. Cochran’s Q, Q df, and Q-test p describe the test for heterogeneity, tau^2^ denotes the estimated between-dataset variance, I^2^ denotes the percentage of total variability attributable to heterogeneity and H^2^ denotes the relative excess in variability due to heterogeneity.

| Section | Statistic | Value |
| --- | --- | --- |
| Pooled effect | Method | REML |
| Pooled effect | Estimate | -0.02 |
| Pooled effect | SE | 0.04 |
| Pooled effect | 95% CI lower | -0.10 |
| Pooled effect | 95% CI upper | 0.05 |
| Pooled effect | z | -0.57 |
| Pooled effect | p | 0.569 |
| Heterogeneity | Cochran’s Q | 1.18 |
| Heterogeneity | Q df | 2 |
| Heterogeneity | Q-test p | 0.555 |
| Heterogeneity | tau^2^ | 0.00 |
| Heterogeneity | I^2^ (%) | 0.00 |
| Heterogeneity | H^2^ | 1.00 |

# Supplementary Note 4: Hypertension PRS

Table N4.1: PRS information for Hypertension PRS

| Metric | Value |
| --- | --- |
| PRS | Hypertension |
| PGS catalog link | <https://www.pgscatalog.org/score/PGS002765> |
| Available PRS SNPs | 1057993 |
| Unavailable PRS SNPs | 19901 |
| SNP missingness | 1.85% |

Table N4.2: Shapiro–Wilk test results to assess normality of Hypertension PRS in the study groups. For each group, the Shapiro–Wilk test statistic W and corresponding P-value are reported.

| Group | W | P-value |
| --- | --- | --- |
| 1000G_eur | 0.99 | 0.028 |
| iRLS_cases | 1.00 | 0.140 |
| uremic_controls | 1.00 | 0.027 |
| uRLS_cases | 1.00 | 0.699 |

Figure N4.1: Group-wise Q-Q plots of Hypertension PRS. Within each group the PRS was scales prior to plotting. The plot allows visual assessment of deviations from normality.

**Hypertension PRS Score by group (in ascending order by median)**

Figure N4.2: Hypertension PRS Score by group in ascending order by median PRS

1000

G_eur

iRLS_cases

uremic_controls

uRLS_cases

−1.0

−0.5

0.0

0.5

PRS

Table N4.3: Welch one-way ANOVA comparing Hypertension PRS across groups. F statistic denotes Welch’s F statistic, Numerator df and Denominator df denote the numerator and denominator degrees of freedom and P-value denotes the corresponding p-value.

| Test | F statistic | Numerator df | Denominator df | P-value |
| --- | --- | --- | --- | --- |
| Welch one-way ANOVA | 2.78 | 3 | 1131.30 | 0.040 |

Table N4.4: Results of pairwise Welch’s t-tests comparing mean Hypertension PRS between groups. Test pair denotes the two groups compared, Mean1 and Mean2 denote the group means for the first and second group in the test pair, Effect denotes the difference Mean1 - Mean2, t denotes the Welch’s t-statistic, 95% CI lower and 95% CI upper denote the bounds of the 95% confidence interval for the mean difference and P-value denotes the corresponding two-sided p-value.

| Test pair | Effect | Mean1 | Mean2 | t | 95% CI lower | 95% CI upper | P-value |
| --- | --- | --- | --- | --- | --- | --- | --- |
| uRLS_cases vs iRLS_cases | 0.03 | 0.03 | -2.27e-03 | 2.27 | 4.40e-03 | 0.06 | 0.024 |
| uremic_controls vs iRLS_cases | -8.88e-05 | -2.36e-03 | -2.27e-03 | -8.07e-03 | -0.02 | 0.02 | 0.994 |
| uRLS_cases vs 1000G_eur | 0.04 | 0.03 | -0.01 | 2.79 | 0.01 | 0.08 | 0.005 |
| uremic_controls vs 1000G_eur | 0.01 | -2.36e-03 | -0.01 | 0.91 | -0.01 | 0.04 | 0.363 |
| uRLS_cases vs uremic_controls | 0.03 | 0.03 | -2.36e-03 | 2.29 | 4.70e-03 | 0.06 | 0.022 |
| iRLS_cases vs 1000G_eur | 0.01 | -2.27e-03 | -0.01 | 0.91 | -0.01 | 0.04 | 0.364 |

Table N4.5: Summary of Hypertension PRS values by RLS status for Hypertension in the GER1 dataset. N is the number of individuals in each group and Mean Hypertension PRS denotes the mean PRS in each group.

| Group | N | Mean Hypertension PRS |
| --- | --- | --- |
| RLS controls | 332 | 6.99e-03 |
| RLS cases | 169 | 0.04 |

Table N4.6: Results of linear regression in the GER1 dataset with Hypertension PRS as outcome and RLS status, sex and age as predictors. 95% CI lower and 95% CI upper are the lower and upper bound of the 95% confidence interval for the regression coefficient Beta. SE is the estimated standard error of Beta, t denotes the t-statistic testing whether the estimated coefficient differs from zero and P-value denotes the corresponding two-sided p-value.

| Variable | Beta | SE | 95% CI lower | 95% CI upper | t | P-value |
| --- | --- | --- | --- | --- | --- | --- |
| (Intercept) | 0.04 | 0.06 | -0.08 | 0.16 | 0.60 | 0.550 |
| RLS_statuscase | 0.03 | 0.02 | -0.01 | 0.07 | 1.36 | 0.176 |
| SEX | -5.76e-03 | 0.02 | -0.05 | 0.04 | -0.27 | 0.788 |
| Age | -3.36e-04 | 8.27e-04 | -1.96e-03 | 1.29e-03 | -0.41 | 0.685 |

Table N4.7: Summary of Hypertension PRS values by RLS status for Hypertension in the GER2 dataset. N is the number of individuals in each group and Mean Hypertension PRS denotes the mean PRS in each group.

| Group | N | Mean Hypertension PRS |
| --- | --- | --- |
| RLS controls | 164 | 0.01 |
| RLS cases | 74 | 0.03 |

Table N4.8: Results of linear regression in the GER2 dataset with Hypertension PRS as outcome and RLS status, sex and age as predictors. 95% CI lower and 95% CI upper are the lower and upper bound of the 95% confidence interval for the regression coefficient Beta. SE is the estimated standard error of Beta, t denotes the t-statistic testing whether the estimated coefficient differs from zero and P-value denotes the corresponding two-sided p-value.

| Variable | Beta | SE | 95% CI lower | 95% CI upper | t | P-value |
| --- | --- | --- | --- | --- | --- | --- |
| (Intercept) | 0.04 | 0.09 | -0.12 | 0.21 | 0.51 | 0.608 |
| RLS_statuscase | 0.02 | 0.03 | -0.04 | 0.08 | 0.67 | 0.502 |
| SEX | -0.08 | 0.03 | -0.14 | -0.01 | -2.37 | 0.018 |
| Age | 1.05e-03 | 1.01e-03 | -9.35e-04 | 3.04e-03 | 1.04 | 0.301 |

Table N4.9: Summary of Hypertension PRS values by RLS status for Hypertension in the GRK dataset. N is the number of individuals in each group and Mean Hypertension PRS denotes the mean PRS in each group.

| Group | N | Mean Hypertension PRS |
| --- | --- | --- |
| RLS controls | 319 | -0.02 |
| RLS cases | 118 | 0.02 |

Table N4.10: Results of linear regression in the GRK dataset with Hypertension PRS as outcome and RLS status, sex and age as predictors. 95% CI lower and 95% CI upper are the lower and upper bound of the 95% confidence interval for the regression coefficient Beta. SE is the estimated standard error of Beta, t denotes the t-statistic testing whether the estimated coefficient differs from zero and P-value denotes the corresponding two-sided p-value.

| Variable | Beta | SE | 95% CI lower | 95% CI upper | t | P-value |
| --- | --- | --- | --- | --- | --- | --- |
| (Intercept) | -0.05 | 0.06 | -0.16 | 0.06 | -0.89 | 0.374 |
| RLS_statuscase | 0.04 | 0.02 | -5.24e-03 | 0.08 | 1.73 | 0.084 |
| SEX | 0.04 | 0.02 | -2.05e-03 | 0.08 | 1.86 | 0.064 |
| Age | -3.46e-04 | 7.60e-04 | -1.84e-03 | 1.14e-03 | -0.46 | 0.649 |

control

case

−0.5

0.0

0.5

**GER1 dataset**

RLS_status

PRS_adj

control

case

−1.0

−0.5

0.0

0.5

**GER2 dataset**

RLS_status

PRS_adj

control

case

−0.6

−0.4

−0.2

0.0

0.2

0.4

**GRK dataset**

RLS_status

PRS_adj

Figure N4.3: Hypertension PRS score by RLS status across the datasets GER1, GER2 and GRK.

Table N4.11: Random-effects meta-analysis of the association between RLS status and Hypertension PRS across the GER1, GER2, and GRK datasets. The meta-analysis used the regression coefficients and standard errors for RLS status from dataset-specific linear regression models with Hypertension PRS as the outcome and RLS status, sex, and age as predictors. The pooled effect was estimated using restricted maximum likelihood (REML). SE denotes the standard error of the pooled estimate; 95% CI lower and 95% CI upper denote the bounds of the 95% confidence interval, z denotes the test statistic for the pooled effect, and p denotes the corresponding p-value. Cochran’s Q, Q df, and Q-test p describe the test for heterogeneity, tau^2^ denotes the estimated between-dataset variance, I^2^ denotes the percentage of total variability attributable to heterogeneity and H^2^ denotes the relative excess in variability due to heterogeneity.

| Section | Statistic | Value |
| --- | --- | --- |
| Pooled effect | Method | REML |
| Pooled effect | Estimate | 0.03 |
| Pooled effect | SE | 0.01 |
| Pooled effect | 95% CI lower | 4.07e-03 |
| Pooled effect | 95% CI upper | 0.06 |
| Pooled effect | z | 2.25 |
| Pooled effect | p | 0.025 |
| Heterogeneity | Cochran’s Q | 0.24 |
| Heterogeneity | Q df | 2 |
| Heterogeneity | Q-test p | 0.888 |
| Heterogeneity | tau^2^ | 0.00 |
| Heterogeneity | I^2^ (%) | 0.00 |
| Heterogeneity | H^2^ | 1.00 |

# Supplementary Note 5: Depression PRS

Table N5.1: PRS information for Depression PRS

| Metric | Value |
| --- | --- |
| PRS | Depression |
| PGS catalog link | <https://www.pgscatalog.org/score/PGS003333> |
| Available PRS SNPs | 1068211 |
| Unavailable PRS SNPs | 20204 |
| SNP missingness | 1.86% |

Table N5.2: Shapiro–Wilk test results to assess normality of Depression PRS in the study groups. For each group, the Shapiro–Wilk test statistic W and corresponding P-value are reported.

| Group | W | P-value |
| --- | --- | --- |
| 1000G_eur | 1.00 | 0.770 |
| iRLS_cases | 1.00 | 0.246 |
| uremic_controls | 1.00 | 0.807 |
| uRLS_cases | 1.00 | 0.374 |

Figure N5.1: Group-wise Q-Q plots of Depression PRS. Within each group the PRS was scales prior to plotting. The plot allows visual assessment of deviations from normality.

**Depression PRS Score by group (in ascending order by median)**

1000

G_eur

uremic_controls

uRLS_cases

iRLS_cases

−0.3

−0.1

0.1

0.2

0.3

Figure N5.2: Depression PRS Score by group in ascending order by median PRS

Table N5.3: Welch one-way ANOVA comparing Depression PRS across groups. F statistic denotes Welch’s F statistic, Numerator df and Denominator df denote the numerator and denominator degrees of freedom and P-value denotes the corresponding p-value.

| Test | F statistic | Numerator df | Denominator df | P-value |
| --- | --- | --- | --- | --- |
| Welch one-way ANOVA | 1.76 | 3 | 1135.62 | 0.154 |

# Supplementary Note 6: Low density lipoprotein measurement PRS

Table N6.1: PRS information for Low density lipoprotein measurement PRS

| Metric | Value |
| --- | --- |
| PRS | Low density lipoprotein measurement |
| PGS catalog link | <https://www.pgscatalog.org/score/PGS003976> |
| Available PRS SNPs | 806757 |
| Unavailable PRS SNPs | 14377 |
| SNP missingness | 1.75% |

Table N6.2: Shapiro–Wilk test results to assess normality of Low density lipoprotein measurement PRS in the study groups. For each group, the Shapiro–Wilk test statistic W and corresponding P-value are reported.

| Group | W | P-value |
| --- | --- | --- |
| 1000G_eur | 1.00 | 0.343 |
| iRLS_cases | 1.00 | 0.678 |
| uremic_controls | 1.00 | 0.692 |
| uRLS_cases | 1.00 | 0.939 |

Figure N6.1: Group-wise Q-Q plots of Low density lipoprotein measurement PRS. Within each group the PRS was scales prior to plotting. The plot allows visual assessment of deviations from normality.

**Low density lipoprotein measurement PRS Score by group (in ascending order by median)**

uremic_controls

1000

G_eur

iRLS_cases

uRLS_cases

−20

0

20

40

Figure N6.2: Low density lipoprotein measurement PRS Score by group in ascending order by median PRS

Table N6.3: Welch one-way ANOVA comparing Low density lipoprotein measurement PRS across groups. F statistic denotes Welch’s F statistic, Numerator df and Denominator df denote the numerator and denominator degrees of freedom and P-value denotes the corresponding p-value.

| Test | F statistic | Numerator df | Denominator df | P-value |
| --- | --- | --- | --- | --- |
| Welch one-way ANOVA | 1.03 | 3 | 1117.24 | 0.379 |

# Supplementary Note 7: Ischemic stroke PRS

Table N7.1: PRS information for Ischemic stroke PRS

| Metric | Value |
| --- | --- |
| PRS | Ischemic stroke |
| PGS catalog link | <https://www.pgscatalog.org/score/PGS000911> |
| Available PRS SNPs | 506082 |
| Unavailable PRS SNPs | 24851 |
| SNP missingness | 4.68% |

Table N7.2: Shapiro–Wilk test results to assess normality of Ischemic stroke PRS in the study groups. For each group, the Shapiro–Wilk test statistic W and corresponding P-value are reported.

| Group | W | P-value |
| --- | --- | --- |
| 1000G_eur | 1.00 | 0.769 |
| iRLS_cases | 1.00 | 0.861 |
| uremic_controls | 1.00 | 0.594 |
| uRLS_cases | 1.00 | 0.549 |

Figure N7.1: Group-wise Q-Q plots of Ischemic stroke PRS. Within each group the PRS was scales prior to plotting. The plot allows visual assessment of deviations from normality.

**Ischemic stroke PRS Score by group (in ascending order by median)**

uRLS_cases

1000

G_eur

iRLS_cases

uremic_controls

−1

0

1

2

Figure N7.2: Ischemic stroke PRS Score by group in ascending order by median PRS

Table N7.3: Welch one-way ANOVA comparing Ischemic stroke PRS across groups. F statistic denotes

Welch’s F statistic, Numerator df and Denominator df denote the numerator and denominator degrees of freedom and P-value denotes the corresponding p-value.

| Test | F statistic | Numerator df | Denominator df | P-value |
| --- | --- | --- | --- | --- |
| Welch one-way ANOVA | 0.46 | 3 | 1148.38 | 0.709 |

# Supplementary Note 8: Heart failure PRS

Table N8.1: PRS information for Heart failure PRS

| Metric | Value |
| --- | --- |
| PRS | Heart failure |
| PGS catalog link | <https://www.pgscatalog.org/score/PGS003969> |
| Available PRS SNPs | 38 |
| Unavailable PRS SNPs | 1 |
| SNP missingness | 2.56% |

Table N8.2: Shapiro–Wilk test results to assess normality of Heart failure PRS in the study groups. For each group, the Shapiro–Wilk test statistic W and corresponding P-value are reported.

| Group | W | P-value |
| --- | --- | --- |
| 1000G_eur | 1.00 | 0.554 |
| iRLS_cases | 1.00 | 0.760 |
| uremic_controls | 1.00 | 0.703 |
| uRLS_cases | 1.00 | 0.893 |

Figure N8.1: Group-wise Q-Q plots of Heart failure PRS. Within each group the PRS was scales prior to plotting. The plot allows visual assessment of deviations from normality.

**Heart failure PRS Score by group (in ascending order by median)**

uremic_controls

uRLS_cases

1000

G_eur

iRLS_cases

−0.6

−0.4

−0.2

0.0

0.2

0.4

0.6

Figure N8.2: Heart failure PRS Score by group in ascending order by median PRS

Table N8.3: Welch one-way ANOVA comparing Heart failure PRS across groups. F statistic denotes Welch’s F statistic, Numerator df and Denominator df denote the numerator and denominator degrees of freedom and P-value denotes the corresponding p-value.

| Test | F statistic | Numerator df | Denominator df | P-value |
| --- | --- | --- | --- | --- |
| Welch one-way ANOVA | 1.64 | 3 | 1142.92 | 0.179 |

# Supplementary Note 9: Coronary artery disease PRS

Table N9.1: PRS information for Coronary artery disease PRS

| Metric | Value |
| --- | --- |
| PRS | Coronary artery disease |
| PGS catalog link | <https://www.pgscatalog.org/score/PGS004595> |
| Available PRS SNPs | 160 |
| Unavailable PRS SNPs | 4 |
| SNP missingness | 2.44% |

Table N9.2: Shapiro–Wilk test results to assess normality of Coronary artery disease PRS in the study groups. For each group, the Shapiro–Wilk test statistic W and corresponding P-value are reported.

| Group | W | P-value |
| --- | --- | --- |
| 1000G_eur | 1.00 | 0.699 |
| iRLS_cases | 1.00 | 0.531 |
| uremic_controls | 1.00 | 0.497 |
| uRLS_cases | 1.00 | 0.654 |

Figure N9.1: Group-wise Q-Q plots of Coronary artery disease PRS. Within each group the PRS was scales prior to plotting. The plot allows visual assessment of deviations from normality.

Figure N9.2: Coronary artery disease PRS Score by group in ascending order by median PRS

Table N9.3: Welch one-way ANOVA comparing Coronary artery disease PRS across groups. F statistic denotes Welch’s F statistic, Numerator df and Denominator df denote the numerator and denominator degrees of freedom and P-value denotes the corresponding p-value.

| Test | F statistic | Numerator df | Denominator df | P-value |
| --- | --- | --- | --- | --- |
| Welch one-way ANOVA | 0.22 | 3 | 1128.40 | 0.883 |

# Supplementary Note 10: Anxiety PRS

Table N10.1: PRS information for Anxiety PRS

| Metric | Value |
| --- | --- |
| PRS | Anxiety |
| PGS catalog link | <https://www.pgscatalog.org/score/PGS004451> |
| Available PRS SNPs | 1040410 |
| Unavailable PRS SNPs | 19529 |
| SNP missingness | 1.84% |

Table N10.2: Shapiro–Wilk test results to assess normality of Anxiety PRS in the study groups. For each group, the Shapiro–Wilk test statistic W and corresponding P-value are reported.

| Group | W | P-value |
| --- | --- | --- |
| 1000G_eur | 1.00 | 0.716 |
| iRLS_cases | 1.00 | 0.547 |
| uremic_controls | 1.00 | 0.229 |
| uRLS_cases | 1.00 | 0.629 |

Figure N10.1: Group-wise Q-Q plots of Anxiety PRS. Within each group the PRS was scales prior to plotting.The plot allows visual assessment of deviations from normality.

Figure N10.2: Anxiety PRS Score by group in ascending order by median PRS

Table N10.3: Welch one-way ANOVA comparing Anxiety PRS across groups. F statistic denotes Welch’s F statistic, Numerator df and Denominator df denote the numerator and denominator degrees of freedom and P-value denotes the corresponding p-value.

| Test | F statistic | Numerator df | Denominator df | P-value |
| --- | --- | --- | --- | --- |
| Welch one-way ANOVA | 2.54 | 3 | 1134.72 | 0.055 |

# Supplementary Note 11: Abdominal aortic aneurism PRS

Table N11.1: PRS information for Abdominal aortic aneurism PRS

| Metric | Value |
| --- | --- |
| PRS | Abdominal aortic aneurism |
| PGS catalog link | <https://www.pgscatalog.org/score/PGS000753> |
| Available PRS SNPs | 29 |
| Unavailable PRS SNPs | 0 |
| SNP missingness | 0% |

Table N11.2: Shapiro–Wilk test results to assess normality of Abdominal aortic aneurism PRS in the study groups. For each group, the Shapiro–Wilk test statistic W and corresponding P-value are reported.

| Group | W | P-value |
| --- | --- | --- |
| 1000G_eur | 1.00 | 0.451 |
| iRLS_cases | 1.00 | 0.694 |
| uremic_controls | 1.00 | 0.869 |
| uRLS_cases | 1.00 | 0.351 |

Figure N11.1: Group-wise Q-Q plots of Abdominal aortic aneurism PRS. Within each group the PRS was scales prior to plotting. The plot allows visual assessment of deviations from normality.

**Abdominal aortic aneurism PRS Score by group (in ascending order by median)**

uRLS_cases

uremic_controls

iRLS_cases

G_eur

1000

−1.5

−1.0

−0.5

0.0

0.5

1.0

Figure N11.2: Abdominal aortic aneurism PRS Score by group in ascending order by median PRS

Table N11.3: Welch one-way ANOVA comparing Abdominal aortic aneurism PRS across groups. F statistic denotes Welch’s F statistic, Numerator df and Denominator df denote the numerator and denominator degrees of freedom and P-value denotes the corresponding p-value.

| Test | F statistic | Numerator df | Denominator df | P-value |
| --- | --- | --- | --- | --- |
| Welch one-way ANOVA | 1.66 | 3 | 1145.57 | 0.173 |

## List of Figures

1 Locus zoom plot of rs36127502 using a 500kb window. LD is based on a European 1000g reference panel.
2 Locus zoom plot of rs5769388 using a 500kb window. LD is based on a European 1000g reference panel. The Ensembl ID of AL078622.1 is ENSG00000286381 and is called the NHIP gene
3 Multidimensional scaling (MDS) plots of the first two components for the three uremic GWAS datasets projected onto individuals from the 1000G project. Panels A–C show GER1, GER2 and GRK respectively, after basic quality control steps but before excluding individuals that deviate more than 3 SD from the mean of any of the first 5 MDS components calculated within dataset without considering 1000G individuals. Panels D–F show the corresponding datasets after outlier exclusion.
4 Panels A–C show scree plots of the eigenvalues for the first ten PCs calculated for GER1, GER2 and GRK, respectively

N1.1	Group-wise Q-Q plots of RLS PRS. Within each group the PRS was scales prior to plotting. The plot allows visual assessment of deviations from normality

N1.2	RLS PRS Score by group in ascending order by median PRS

N1.3	RLS PRS score by RLS status across the datasets GER1, GER2 and GRK

N2.1	Group-wise Q-Q plots of RLS PRS. Within each group the PRS was scales prior to plotting. Uremic groups are without individuals with known familial RLS. The plot allows visual assessment of deviations from normality.

N2.2	RLS PRS Score by group in ascending order by median PRS. Uremic groups are without individuals with known familial RLS.

N2.3	RLS PRS score by RLS status across the datasets GER1, GER2 and GRK. Individuals with known familial RLS were removed.

N3.1	Group-wise Q-Q plots of Type 2 diabetes PRS. Within each group the PRS was scales prior to plotting. The plot allows visual assessment of deviations from normality

N3.2	Type 2 diabetes PRS Score by group in ascending order by median PRS

N3.3	Type 2 diabetes PRS score by RLS status across the datasets GER1, GER2 and GRK

N4.1	Group-wise Q-Q plots of Hypertension PRS. Within each group the PRS was scales prior to plotting. The plot allows visual assessment of deviations from normality

N4.2	Hypertension PRS Score by group in ascending order by median PRS

N4.3	Hypertension PRS score by RLS status across the datasets GER1, GER2 and GRK.

N5.1	Group-wise Q-Q plots of Depression PRS. Within each group the PRS was scales prior to plotting. The plot allows visual assessment of deviations from normality.

N5.2	Depression PRS Score by group in ascending order by median PRS

N6.1	Group-wise Q-Q plots of Low density lipoprotein measurement PRS. Within each group the PRS was scales prior to plotting. The plot allows visual assessment of deviations from normality.

N6.2	Low density lipoprotein measurement PRS Score by group in ascending order by median PRS

N7.1	Group-wise Q-Q plots of Ischemic stroke PRS. Within each group the PRS was scales prior to plotting. The plot allows visual assessment of deviations from normality.

N7.2	Ischemic stroke PRS Score by group in ascending order by median PRS

N8.1	Group-wise Q-Q plots of Heart failure PRS. Within each group the PRS was scales prior to plotting. The plot allows visual assessment of deviations from normality

N8.2	Heart failure PRS Score by group in ascending order by median PRS

N9.1	Group-wise Q-Q plots of Coronary artery disease PRS. Within each group the PRS was scales prior to plotting. The plot allows visual assessment of deviations from normality

N9.2	Coronary artery disease PRS Score by group in ascending order by median PRS

N10.1	Group-wise Q-Q plots of Anxiety PRS. Within each group the PRS was scales prior to plotting. The plot allows visual assessment of deviations from normality

N10.2	Anxiety PRS Score by group in ascending order by median PRS

N11.1	Group-wise Q-Q plots of Abdominal aortic aneurism PRS. Within each group the PRS was scales prior to plotting. The plot allows visual assessment of deviations from normality

N11.2	Abdominal aortic aneurism PRS Score by group in ascending order by median PRS

**List of Tables**

N1.1	PRS information for RLS PRS .

N1.2	Shapiro–Wilk test results to assess normality of RLS PRS in the study groups. For each group, the Shapiro–Wilk test statistic W and corresponding P-value are reported

N1.3	Welch one-way ANOVA comparing RLS PRS across groups. F statistic denotes Welch’s F statistic, Numerator df and Denominator df denote the numerator and denominator degrees of freedom and P-value denotes the corresponding p-value.

N1.4	Results of pairwise Welch’s t-tests comparing mean RLS PRS between groups. Test pair denotes the two groups compared, Mean1 and Mean2 denote the group means for the first and second group in the test pair, Effect denotes the difference Mean1 - Mean2, t denotes the Welch’s t-statistic, 95% CI lower and 95% CI upper denote the bounds of the 95% confidence interval for the mean difference and P-value denotes the corresponding two-sided p-value.

N1.5	Summary of RLS PRS values by RLS status for RLS in the GER1 dataset. N is the numberof individuals in each group and Mean RLS PRS denotes the mean PRS in each group.

N1.6	Results of linear regression in the GER1 dataset with RLS PRS as outcome and RLS status, sex and age as predictors. 95% CI lower and 95% CI upper are the lower and upper bound of the 95% confidence interval for the regression coefficient Beta. SE is the estimated standard error of Beta, t denotes the t-statistic testing whether the estimated coefficient differs from zero and P-value denotes the corresponding two-sided p-value.

N1.7	Summary of RLS PRS values by RLS status for RLS in the GER2 dataset. N is the number of individuals in each group and Mean RLS PRS denotes the mean PRS in each group.

N1.8	Results of linear regression in the GER2 dataset with RLS PRS as outcome and RLS status, sex and age as predictors. 95% CI lower and 95% CI upper are the lower and upper bound of the 95% confidence interval for the regression coefficient Beta. SE is the estimated standard error of Beta, t denotes the t-statistic testing whether the estimated coefficient differs from zero and P-value denotes the corresponding two-sided p-value.

N1.9	Summary of RLS PRS values by RLS status for RLS in the GRK dataset. N is the number of individuals in each group and Mean RLS PRS denotes the mean PRS in each group.

N1.10	Results of linear regression in the GRK dataset with RLS PRS as outcome and RLS status, sex and age as predictors. 95% CI lower and 95% CI upper are the lower and upper bound of the 95% confidence interval for the regression coefficient Beta. SE is the estimated standard error of Beta, t denotes the t-statistic testing whether the estimated coefficient differs from zero and P-value denotes the corresponding two-sided p-value.

N1.11	Random-effects meta-analysis of the association between RLS status and RLS PRS across the GER1, GER2, and GRK datasets. The meta-analysis used the regression coefficients and standard errors for RLS status from dataset-specific linear regression models with RLS PRS as the outcome and RLS status, sex, and age as predictors. The pooled effect was estimated using restricted maximum likelihood (REML). SE denotes the standard error of the pooled estimate; 95% CI lower and 95% CI upper denote the bounds of the 95% confidence interval, z denotes the test statistic for the pooled effect, and p denotes the corresponding p-value. Cochran’s Q, Q df, and Q-test p describe the test for heterogeneity, tau^2^ denotes the estimated between-dataset variance, I^2^ denotes the percentage of total variability attributable to heterogeneity and H^2^ denotes the relative excess in variability due to heterogeneity.

N2.1	PRS information for RLS PRS.

N2.2	Shapiro–Wilk test results to assess normality of RLS PRS in the study groups. Uremic groups are without individuals with known familial RLS. For each group, the Shapiro–Wilk test statistic W and corresponding P-value are reported.

N2.3	Welch one-way ANOVA comparing RLS PRS across groups. Uremic groups are without individuals with known familial RLS. F statistic denotes Welch’s F statistic, Numerator df and Denominator df denote the numerator and denominator degrees of freedom and P-value denotes the corresponding p-value.

N2.4	Results of pairwise Welch’s t-tests comparing mean RLS PRS between groups. Uremic groups are without individuals with known familial RLS. Test pair denotes the two groups compared, Mean1 and Mean2 denote the group means for the first and second group in the test pair, Effect denotes the difference Mean1 - Mean2, t denotes the Welch’s t-statistic, 95% CI lower and 95% CI upper denote the bounds of the 95% confidence interval for the mean difference and P-value denotes the corresponding two-sided p-value.

N2.5	Summary of RLS PRS values by RLS status in the GER1 dataset. Individuals with known familial RLS were removed. N is the number of individuals in each group and Mean RLS PRS denotes the mean PRS in each group.

N2.6	Results of linear regression in the GER1 dataset with RLS PRS as outcome and RLS status, sex and age as predictors. Individuals with known familial RLS were removed. 95% CI lower and 95% CI upper are the lower and upper bound of the 95% confidence interval for the regression coefficient Beta. SE is the estimated standard error of Beta, t denotes the t-statistic testing whether the estimated coefficient differs from zero and P-value denotes the corresponding two-sided p-value.

N2.7	Summary of RLS PRS values by RLS status in the GER2 dataset. Individuals with known familial RLS were removed. N is the number of individuals in each group and Mean RLS PRS denotes the mean PRS in each group.

N2.8	Results of linear regression in the GER2 dataset with RLS PRS as outcome and RLS status, sex and age as predictors. Individuals with known familial RLS were removed. 95% CI lower and 95% CI upper are the lower and upper bound of the 95% confidence interval for the regression coefficient Beta. SE is the estimated standard error of Beta, t denotes the t-statistic testing whether the estimated coefficient differs from zero and P-value denotes the corresponding two-sided p-value.

N2.9	Summary of RLS PRS values in the GRK dataset. Individuals with known familial RLS were removed. N is the number of individuals in each group and Mean RLS PRS denotes the mean PRS in each group.

N2.10	Results of linear regression in the GRK dataset with RLS PRS as outcome and RLS status, sex and age as predictors. Individuals with known familial RLS were removed. 95% CI lower and 95% CI upper are the lower and upper bound of the 95% confidence interval for the regression coefficient Beta. SE is the estimated standard error of Beta, t denotes the t-statistic testing whether the estimated coefficient differs from zero and P-value denotes the corresponding two-sided p-value.

N2.11	Random-effects meta-analysis of the association between RLS status and RLS PRS across the GER1, GER2, and GRK datasets. Individuals with known familial RLS were removed. The meta-analysis used the regression coefficients and standard errors for RLS status from dataset-specific linear regression models with RLS without familial cases PRS as the outcome and RLS status, sex, and age as predictors. The pooled effect was estimated using restricted maximum likelihood (REML). SE denotes the standard error of the pooled estimate; 95% CI lower and 95% CI upper denote the bounds of the 95% confidence interval, z denotes the test statistic for the pooled effect, and p denotes the corresponding p-value. Cochran’s Q, Q df, and Q-test p describe the test for heterogeneity, tau^2^ denotes the estimated between-dataset variance, I^2^ denotes the percentage of total variability attributable to heterogeneity and H^2^ denotes the relative excess in variability due to heterogeneity.

N3.1	PRS information for Type 2 diabetes PRS.

N3.2	Shapiro–Wilk test results to assess normality of Type 2 diabetes PRS in the study groups. For each group, the Shapiro–Wilk test statistic W and corresponding P-value are reported.

N3.3	Welch one-way ANOVA comparing Type 2 diabetes PRS across groups. F statistic denotes Welch’s F statistic, Numerator df and Denominator df denote the numerator and denominator degrees of freedom and P-value denotes the corresponding p-value.

N3.4	Results of pairwise Welch’s t-tests comparing mean Type 2 diabetes PRS between groups. Test pair denotes the two groups compared, Mean1 and Mean2 denote the group means for the first and second group in the test pair, Effect denotes the difference Mean1 - Mean2, t denotes the Welch’s t-statistic, 95% CI lower and 95% CI upper denote the bounds of the 95% confidence interval for the mean difference and P-value denotes the corresponding two-sided p-value.

N3.5	Summary of Type 2 diabetes PRS values by RLS status for Type 2 diabetes in the GER1 dataset. N is the number of individuals in each group and Mean Type 2 diabetes PRS denotes the mean PRS in each group.

N3.6	Results of linear regression in the GER1 dataset with Type 2 diabetes PRS as outcome and RLS status, sex and age as predictors. 95% CI lower and 95% CI upper are the lower and upper bound of the 95% confidence interval for the regression coefficient Beta. SE is the estimated standard error of Beta, t denotes the t-statistic testing whether the estimated coefficient differs from zero and P-value denotes the corresponding two-sided p-value.

N3.7	Summary of Type 2 diabetes PRS values by RLS status for Type 2 diabetes in the GER2 dataset. N is the number of individuals in each group and Mean Type 2 diabetes PRS denotes the mean PRS in each group.

N3.8	Results of linear regression in the GER2 dataset with Type 2 diabetes PRS as outcome and RLS status, sex and age as predictors. 95% CI lower and 95% CI upper are the lower and upper bound of the 95% confidence interval for the regression coefficient Beta. SE is the estimated standard error of Beta, t denotes the t-statistic testing whether the estimated coefficient differs from zero and P-value denotes the corresponding two-sided p-value.

N3.9	Summary of Type 2 diabetes PRS values by RLS status for Type 2 diabetes in the GRK dataset. N is the number of individuals in each group and Mean Type 2 diabetes PRS denotes the mean PRS in each group.

N3.10	Results of linear regression in the GRK dataset with Type 2 diabetes PRS as outcome and RLS status, sex and age as predictors. 95% CI lower and 95% CI upper are the lower and upper bound of the 95% confidence interval for the regression coefficient Beta. SE is the estimated standard error of Beta, t denotes the t-statistic testing whether the estimated coefficient differs from zero and P-value denotes the corresponding two-sided p-value.

N3.11	Random-effects meta-analysis of the association between RLS status and Type 2 diabetes PRS across the GER1, GER2, and GRK datasets. The meta-analysis used the regression coefficients and standard errors for RLS status from dataset-specific linear regression models with Type 2 diabetes PRS as the outcome and RLS status, sex, and age as predictors. The pooled effect was estimated using restricted maximum likelihood (REML). SE denotes the standard error of the pooled estimate; 95% CI lower and 95% CI upper denote the bounds of the 95% confidence interval, z denotes the test statistic for the pooled effect, and p denotes the corresponding p-value. Cochran’s Q, Q df, and Q-test p describe the test for heterogeneity, tau^2^ denotes the estimated between-dataset variance, I^2^ denotes the percentage of total variability attributable to heterogeneity and H^2^ denotes the relative excess in variability due to heterogeneity.

N4.1	PRS information for Hypertension PRS.

N4.2	Shapiro–Wilk test results to assess normality of Hypertension PRS in the study groups. For each group, the Shapiro–Wilk test statistic W and corresponding P-value are reported.

N4.3	Welch one-way ANOVA comparing Hypertension PRS across groups. F statistic denotes Welch’s F statistic, Numerator df and Denominator df denote the numerator and denominator degrees of freedom and P-value denotes the corresponding p-value.

N4.4	Results of pairwise Welch’s t-tests comparing mean Hypertension PRS between groups. Test pair denotes the two groups compared, Mean1 and Mean2 denote the group means for the first and second group in the test pair, Effect denotes the difference Mean1 - Mean2, t denotes the Welch’s t-statistic, 95% CI lower and 95% CI upper denote the bounds of the 95% confidence interval for the mean difference and P-value denotes the corresponding two-sided p-value.

N4.5	Summary of Hypertension PRS values by RLS status for Hypertension in the GER1 dataset. N is the number of individuals in each group and Mean Hypertension PRS denotes the mean PRS in each group.

N4.6	Results of linear regression in the GER1 dataset with Hypertension PRS as outcome and RLS status, sex and age as predictors. 95% CI lower and 95% CI upper are the lower and upper bound of the 95% confidence interval for the regression coefficient Beta. SE is the estimated standard error of Beta, t denotes the t-statistic testing whether the estimated coefficient differs from zero and P-value denotes the corresponding two-sided p-value.

N4.7	Summary of Hypertension PRS values by RLS status for Hypertension in the GER2 dataset. N is the number of individuals in each group and Mean Hypertension PRS denotes the mean PRS in each group.

N4.8	Results of linear regression in the GER2 dataset with Hypertension PRS as outcome and RLS status, sex and age as predictors. 95% CI lower and 95% CI upper are the lower and upper bound of the 95% confidence interval for the regression coefficient Beta. SE is the estimated standard error of Beta, t denotes the t-statistic testing whether the estimated coefficient differs from zero and P-value denotes the corresponding two-sided p-value.

N4.9	Summary of Hypertension PRS values by RLS status for Hypertension in the GRK dataset. N is the number of individuals in each group and Mean Hypertension PRS denotes the mean PRS in each group.

N4.10	Results of linear regression in the GRK dataset with Hypertension PRS as outcome and RLS status, sex and age as predictors. 95% CI lower and 95% CI upper are the lower and upper bound of the 95% confidence interval for the regression coefficient Beta. SE is the estimated standard error of Beta, t denotes the t-statistic testing whether the estimated coefficient differs from zero and P-value denotes the corresponding two-sided p-value.

N4.11	Random-effects meta-analysis of the association between RLS status and Hypertension PRS across the GER1, GER2, and GRK datasets. The meta-analysis used the regression coefficients and standard errors for RLS status from dataset-specific linear regression models with Hypertension PRS as the outcome and RLS status, sex, and age as predictors. The pooled effect was estimated using restricted maximum likelihood (REML). SE denotes the standard error of the pooled estimate; 95% CI lower and 95% CI upper denote the bounds of the 95% confidence interval, z denotes the test statistic for the pooled effect, and p denotes the corresponding p-value. Cochran’s Q, Q df, and Q-test p describe the test for heterogeneity, tau^2^ denotes the estimated between-dataset variance, I^2^ denotes the percentage of total variability attributable to heterogeneity and H^2^ denotes the relative excess in variability due to heterogeneity.

N5.1	PRS information for Depression PRS.

N5.2	Shapiro–Wilk test results to assess normality of Depression PRS in the study groups. For each group, the Shapiro–Wilk test statistic W and corresponding P-value are reported.

N5.3	Welch one-way ANOVA comparing Depression PRS across groups. F statistic denotes Welch’s F statistic, Numerator df and Denominator df denote the numerator and denominator degree of freedom and P-value denotes the corresponding p-value.

N6.1	PRS information for Low density lipoprotein measurement PRS.

N6.2	Shapiro–Wilk test results to assess normality of Low density lipoprotein measurement PRS in the study groups. For each group, the Shapiro–Wilk test statistic W and corresponding P-value are reported.

N6.3	Welch one-way ANOVA comparing Low density lipoprotein measurement PRS across groups. F statistic denotes Welch’s F statistic, Numerator df and Denominator df denote the numerator and denominator degrees of freedom and P-value denotes the corresponding p-value.

N7.1	PRS information for Ischemic stroke PRS.

N7.2	Shapiro–Wilk test results to assess normality of Ischemic stroke PRS in the study groups. For each group, the Shapiro–Wilk test statistic W and corresponding P-value are reported.

N7.3	Welch one-way ANOVA comparing Ischemic stroke PRS across groups. F statistic denotes Welch’s F statistic, Numerator df and Denominator df denote the numerator and denominator degrees of freedom and P-value denotes the corresponding p-value.

N8.1	PRS information for Heart failure PRS.

N8.2	Shapiro–Wilk test results to assess normality of Heart failure PRS in the study groups. For each group, the Shapiro–Wilk test statistic W and corresponding P-value are reported.

N8.3	Welch one-way ANOVA comparing Heart failure PRS across groups. F statistic denotes Welch’s F statistic, Numerator df and Denominator df denote the numerator and denominator degrees of freedom and P-value denotes the corresponding p-value.

N9.1	PRS information for Coronary artery disease PRS.

N9.2	Shapiro–Wilk test results to assess normality of Coronary artery disease PRS in the study groups. For each group, the Shapiro–Wilk test statistic W and corresponding P-value are reported.

N9.3	Welch one-way ANOVA comparing Coronary artery disease PRS across groups. F statistic denotes Welch’s F statistic, Numerator df and Denominator df denote the numerator and denominator degrees of freedom and P-value denotes the corresponding p-value.

N10.1	PRS information for Anxiety PRS.

N10.2	Shapiro–Wilk test results to assess normality of Anxiety PRS in the study groups. For each group, the Shapiro–Wilk test statistic W and corresponding P-value are reported.

N10.3	Welch one-way ANOVA comparing Anxiety PRS across groups. F statistic denotes Welch’s F statistic, Numerator df and Denominator df denote the numerator and denominator degrees of freedom and P-value denotes the corresponding p-value.

N11.1	PRS information for Abdominal aortic aneurism PRS.

N11.2	Shapiro–Wilk test results to assess normality of Abdominal aortic aneurism PRS in the study groups. For each group, the Shapiro–Wilk test statistic W and corresponding P-value are reported.

N11.3	Welch one-way ANOVA comparing Abdominal aortic aneurism PRS across groups. F statistic denotes Welch’s F statistic, Numerator df and Denominator df denote the numerator and denominator degrees of freedom and P-value denotes the corresponding p-value.
